# Supplementary material for: Determining the distance patterns in the movements of future doctors in UK between 2002 and 2015: a retrospective cohort study
Source: BMJ Open. 2024 Feb 29;14(3):e077635. doi: 10.1136/bmjopen-2023-077635 (PMC10973690; doi:10.1136/bmjopen-2023-077635)
Supplement: Supplementary data [file bmjopen-2023-077635supp001.pdf]

Determining the distance patterns in the movements of future doctors in UK between 2002 and 2015: a retrospective cohort study

Lucy A. Hitchings, Benjamin G. Fleet, Daniel Smith, Jonathan Read, Colin Melville, Luigi Sedda

Supplementary File

Contents

Supplementary information 1: Table S1. Cohort of medical students and junior doctors considered int the study. Foundation year one (F1) and Specialty year one (S1) numbers are referred to individuals that started the medical school between 2002 and 2015. 2
Supplementary information 2: Table S2. Number of junior doctors starting S1 between 2011 and 2015 (second row) and contribution by medical school year. 3
Supplementary information 3: A simplified stage only Leslie Matrix 4
Supplementary information 4: UKMED Ethnicity classification 5
Supplementary information 5: Principal component analysis results 6
Supplementary information 6: Lorenz curve 8

Supplementary information 1: Table S1. Cohort of medical students and junior doctors considered int the study. Foundation year one (F1) and Specialty year one (S1) numbers are referred to individuals that started the medical school between 2002 and 2015.

Year	Number medical students starting medical school	Number of junior doctors in F1	Number of junior doctors in S1
2002	6640	0	0
2003	6824	0	0
2004	7190	0	0
2005	7201	0	0
2006	7145	0	0
2007	7042	0	0
2008	7193	0	0
2009	7100	4487	0
2010	7130	5331	0
2011	7193	5684	137
2012	6936	6494	309
2013	6751	6378	236
2014	6810	6752	114
2015	6777	6597	75
Totals	*97932*	*41723*	*871*

Supplementary information 2: Table S2. Number of junior doctors starting S1 between 2011 and 2015 (second row) and contribution by medical school year.

Year junior doctors started S1	2011	2012	2013	2014	2015
Number of junior doctors in S1	137	309	236	114	75
Proportion (in %) of junior doctors in S1 (second row) that started medical school in 2002	14.39	3.39	2.65	2.75	2.78
Proportion (in %) of junior doctors in S1 (second row) that started medical school in 2003	75.76	29.49	11.95	14.68	6.94
Proportion (in %) of junior doctors in S1 (second row) that started medical school in 2004	6.82	58.98	18.14	28.44	8.33
Proportion (in %) of junior doctors in S1 (second row) that started medical school in 2005	2.27	7.12	63.72	26.61	31.94
Proportion (in %) of junior doctors in S1 (second row) that started medical school in 2006	0.76	0.68	2.21	23.85	20.83
Proportion (in %) of junior doctors in S1 (second row) that started medical school in 2007	0.00	0.34	1.33	0.00	22.22
Proportion (in %) of junior doctors in S1 (second row) that started medical school in 2008	0.00	0.00	0.00	0.92	3.39
Proportion (in %) of junior doctors in S1 (second row) that started medical school in 2009	0.00	0.00	0.00	2.75	3.57

Supplementary information 3: A simplified stage only Leslie Matrix

A distance matrix is a square matrix that consists of the distances between elements, where the elements are the three stages required to complete the medical career. Those three stages are medical school, foundation training and specialty training. Movements are defined as the distance covered by the students from and to postcodes of two successive stages. Movement distances are lagged at 25 km interval.

The following example describes the construction of the Leslie matrix for the first three distances (D1=0-25km, D2=25-50km and D3=50-75km). First consider students travelling from family home to medical school, they are grouped by distance along the matrix diagonal (matrix A):

$$A = \begin{bmatrix} D_1 & & \\ & D_2 & \\ & & D_3 \end{bmatrix}$$

For each of the students who travelled a particular distance onto medical school, each of these will also have moved a certain distance onto their respective foundation training. Starting from the students in D1, they are split between students that travelled 0-25 km (F1), 25-50 km (F2) and 50-75 km (F3). In the same way for students in D2 and D3 (matrices B).

$$B(D_1) = \begin{bmatrix} F_{1,1_{D_1}} & F_{1,2_{D_1}} & F_{1,3_{D,1}} \\ F_{2,1_{D_1}} & & \\ F_{3,1_{D_1}} & & \end{bmatrix}, \quad B(D_2) = \begin{bmatrix} & F_{1,2_{D_2}} & \\ F_{2,1_{D_2}} & F_{2,2_{D_2}} & F_{2,3_{D_2}} \\ & F_{3,2_{D_2}} & \end{bmatrix},$$

$$B(D_3) = \begin{bmatrix} & & F_{1,3_{D_3}} \\ & & F_{2,3_{D_3}} \\ F_{3,1_{D_3}} & F_{3,2_{D_3}} & F_{3,3_{D_3}} \end{bmatrix}$$

Since the matrices are symmetric, $F_{1,2}$ and $F_{2,1}$ have the same values. The same matrices can be created for the F1 distance students going to specialty training (C matrices):

$$C(F_{1,1}) = \begin{bmatrix} S_{1,1_{F1,1}} & S_{1,2_{F1,1}} & S_{1,3_{F1,1}} \\ S_{2,1_{F1,1}} & & \\ S_{3,1_{F1,1}} & & \end{bmatrix}$$

Where $S_{1,1}$ is the number of students that travelled 0-25km in all the stages, i.e. from home to medical school (D1), from medical school to foundation (F1) and from foundation to specialty training (S1). Finally, the Leslie matrix is the sum of matrix A with matrices B and C. This matrix is a symmetric matrix with positive entries and has the same structure as a Leslie matrix, although a Leslie matrix is formulated based on divisions such as stage and age, assuming only population flows in adjacent cells of the matrix. By considering only non-overlapping stages (Murray and Sandercock, 2020), flow to non-contiguous cells is possible (Usher and Williamson, 1970).

References

Murray, Dennis L., and Brett K. Sandercock, eds. Population ecology in practice. John Wiley & Sons, 2020.

Usher, M. B., and M. H. Williamson. "A deterministic matrix model for handling the birth, death, and migration processes of spatially distributed populations." Biometrics (1970): 1-12.

Supplementary information 4: UKMED Ethnicity classification

Level 1 (bold) and Level 2 Ethnicity.
https://www.ethnicity-facts-figures.service.gov.uk/style-guide/ethnic-groups

Asian or Asian British

- Indian
- Pakistani
- Bangladeshi
- Chinese
- Any other Asian background

Black, African, Caribbean or Black British

- African
- Caribbean
- Any other Black, African or Caribbean background

Mixed or multiple ethnic groups

- White and Black Caribbean
- White and Black African
- White and Asian
- Any other Mixed or multiple ethnic background

White

- British (English, Welsh, Scottish, Northern Irish)
- Irish
- Gypsy or Irish Traveller
- Any other White background

Other ethnic group

- Arab
- Any other ethnic group

Supplementary information 5: Principal component analysis results

Table S3: Proportion of variance explained by the distance categories for all years and by each year from principal component analysis applied to all years and yearly Leslie matrices respectively.

km	Overall	2002	2003	2004	2005	2006	2007	2008	2009
0-25	40.98%	42.70%	40.07%	43.12%	45.34%	31.28%	28.55%	28.19%	43.45%
25-50	13.19%	1.32%	12.17%	15.84%	15.87%	10.82%	5.58%	4.72%	14.91%
50-75	7.84%	5.27%	6.47%	9.07%	9.83%	2.62%	6.01%	0%	0%
75-100	4.75%	5.09%	4.98%	5.25%	2.94%	8.12%	7.48%	6.17%	0%
100-125	8.47%	11.15%	7.34%	7.73%	8.36%	6.91%	12.70%	21.80%	0%
125-150	6.00%	9.83%	7.06%	3.07%	3.78%	15.86%	14.69%	7.72%	10.68%
150-175	1.11%	1.50%	1.29%	0.45%	1.23%	4.31%	1.11%	<0.1%	0%
175-200	1.35%	2.39%	1.61%	1.24%	0.37%	0.76%	2.53%	1.54%	6.69%
200-225	0.74%	0%	0.72%	0.57%	0.28%	2.01%	0.49%	4.64%	0%
225-250	1.17%	5.95%	2.06%	1.62%	0.64%	1.62%	0%	1.46%	4.59%
250-275	0.51%	2.26%	0.77%	0.21%	0.23%	0.51%	1.11%	1.13%	1.81%
275-300	0.36%	0%	0.72%	<0.1%	0.59%	0%	0%	0%	0%
300-325	1.34%	0.17%	3.97%	0.22%	0.63%	0.14%	1.15%	0.23%	0%
325-350	0.42%	1.20%	0.46%	0.40%	0.22%	<0.1%	1.11%	0.15%	0%
350-375	1.50%	1.36%	2.26%	1.69%	0.61%	3.88%	0%	3.08%	0%
375-400	0.78%	0%	1.24%	1.15%	0.43%	0%	2.18%	<0.1%	0%
400-425	0.56%	0%	1.01%	0.39%	0.41%	2.19%	<0.1%	1.07%	0%
425-450	0.58%	0%	0.44%	0.58%	0.78%	2.16%	0%	<0.1%	0%
450-475	4.43%	4.60%	2.32%	5.07%	4.20%	2.42%	5.66%	1.93%	0%
475-500	0.36%	1.19%	0.51%	0.26%	0.30%	1.01%	0%	1.48%	0%
500-525	0.33%	1.65%	0.27%	<0.1%	0.20%	0%	0%	1.47%	0%
525-550	<0.1%	0%	<0.1%	<0.1%	<0.1%	0.57%	1.07%	<0.1%	0%
550-575	0.27%	0%	0%	0%	0.20%	0.41%	4.04%	0.68%	0%
575-600	0.22%	0%	0.43%	<0.1%	0.14%	0.99%	0%	0%	0%
600-625	0.14%	0%	0%	0.21%	0.28%	0%	0%	0%	0%
625-650	<0.1%	0%	0%	0.18%	<0.1%	0%	1.08%	0%	0%
650-675	0.16%	0%	0.21%	<0.1%	0.20%	<0.1%	1.07%	0%	0%
675-700	0.11%	<0.1%	0%	<0.1%	0.20%	0%	0%	0%	7.90%
700-725	0.26%	1.13%	<0.1%	<0.1%	0.26%	0%	0.99%	1.39%	0%
725-750	0.42%	0%	0.65%	0.23%	0.41%	0%	1.10%	6.30%	0%
750-775	0.18%	0%	<0.1%	<0.1%	0.58%	<0.1%	0%	<0.1%	0%

775-800	0.71%	1.20%	0.44%	0.97%	<0.1%	<0.1%	0.23%	2.46%	0%
800-825	0.13%	0%	0%	0.19%	0.26%	0%	<0.1%	0%	0%
825-850	0.17%	0%	0.24%	0.18%	<0.1%	0%	0%	0%	0%
850-875	0.18%	0%	0%	0%	0.20%	0%	<0.1%	0%	9.97%
875-900	<0.1%	0%	<0.1%	0%	0%	1.34%	0%	2.15%	0%
900-925	<0.1%	<0.1%	0%	0%	0%	0%	0%	0%	0%
925-950	0%	0%	0%	0%	0%	0%	0%	0%	0%
950-975	<0.1%	0%	0.20%	0%	0%	0%	0%	0%	0%

Supplementary information 6: Lorenz curve

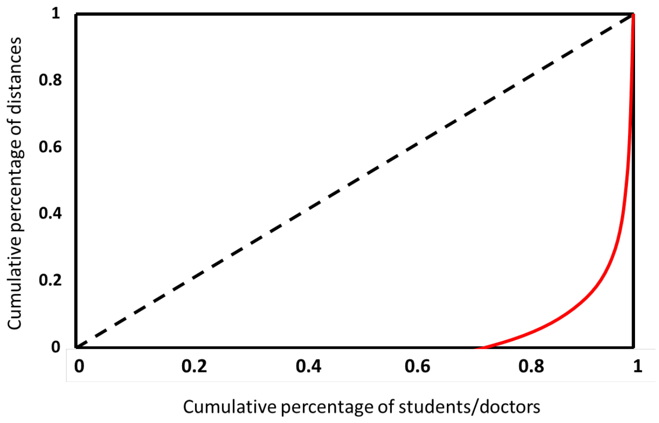

Figure S1: Lorenz curve of the Leslie Matrix. Equality (Gini coefficient equal to 0) is achieved when the Lorenz curve (red line) coincide with the diagonal (dashed). The diagonal represents the status where all distances have equal number of students. In this figure a strong inequality is shown, with the entire Lorenz curve laying on values above 0.7.
