# Supplementary material for: Determining the distance patterns in the movements of future doctors in UK between 2002 and 2015: a retrospective cohort study
Source: BMJ Open. 2024 Feb 29;14(3):e077635. doi: 10.1136/bmjopen-2023-077635 (PMC10973690; doi:10.1136/bmjopen-2023-077635)
Supplement: Author's manuscript [file bmjopen-2023-077635.draft_revisions.pdf]

BMJ Open is committed to open peer review. As part of this commitment we make the peer review history of every article we publish publicly available.

When an article is published we post the peer reviewers' comments and the authors' responses online. We also post the versions of the paper that were used during peer review. These are the versions that the peer review comments apply to.

The versions of the paper that follow are the versions that were submitted during the peer review process. They are not the versions of record or the final published versions. They should not be cited or distributed as the published version of this manuscript.

BMJ Open is an open access journal and the full, final, typeset and author-corrected version of record of the manuscript is available on our site with no access controls, subscription charges or pay-per-view fees (http://bmjopen.bmj.com).

If you have any questions on BMJ Open's open peer review process please email info.bmjopen@bmj.com

BMJ Open

From family home to training: the movements of future doctors in UK between 2002 and 2015.

Journal:	*BMJ Open*
Manuscript ID	bmjopen-2023-077635
Article Type:	Original research
Date Submitted by the Author:	10-Jul-2023
Complete List of Authors:	Hitchings, Lucy A.; Lancaster University, Mathematics and Statistics<br>Fleet, Ben; Lancaster University, Medical School<br>Smith, Daniel; General Medical Council<br>Read, Jonathan; Lancaster University Faculty of Health and Medicine, Centre for Health Informatics, Computing and Statistics, Lancaster Medical School<br>Melville, Colin; The University of Manchester; General Medical Council Manchester<br>Sedda , Luigi ; Lancaster University
Keywords:	HEALTH SERVICES ADMINISTRATION & MANAGEMENT, EDUCATION & TRAINING (see Medical Education & Training), STATISTICS & RESEARCH METHODS

SCHOLARONE™
Manuscripts

I, the Submitting Author has the right to grant and does grant on behalf of all authors of the Work (as defined in the below author licence), an exclusive licence and/or a non-exclusive licence for contributions from authors who are: i) UK Crown employees; ii) where BMJ has agreed a CC-BY licence shall apply, and/or iii) in accordance with the terms applicable for US Federal Government officers or employees acting as part of their official duties; on a worldwide, perpetual, irrevocable, royalty-free basis to BMJ Publishing Group Ltd ("BMJ") its licensees and where the relevant Journal is co-owned by BMJ to the co-owners of the Journal, to publish the Work in this journal and any other BMJ products and to exploit all rights, as set out in our licence.

The Submitting Author accepts and understands that any supply made under these terms is made by BMJ to the Submitting Author unless you are acting as an employee on behalf of your employer or a postgraduate student of an affiliated institution which is paying any applicable article publishing charge ("APC") for Open Access articles. Where the Submitting Author wishes to make the Work available on an Open Access basis (and intends to pay the relevant APC), the terms of reuse of such Open Access shall be governed by a Creative Commons licence – details of these licences and which Creative Commons licence will apply to this Work are set out in our licence referred to above.

Other than as permitted in any relevant BMJ Author's Self Archiving Policies, I confirm this Work has not been accepted for publication elsewhere, is not being considered for publication elsewhere and does not duplicate material already published. I confirm all authors consent to publication of this Work and authorise the granting of this licence.

For peer review only

From family home to training: the movements of future doctors in UK between 2002 and 2015.

Lucy A. Hitchings[1,$,#], Benjamin G. Fleet[2,#], Daniel Smith[3&], Jonathan Read[4&], Colin Melville[3&], Luigi Sedda[5&*]

[1] Mathematics and Statistics, Lancaster University, Lancaster, LA1 4YG, UK.
[2] Lancaster Medical School, Lancaster University, Lancaster, LA1 4YG, UK.
[3] General Medical Council, Manchester, M3 3AW, UK.
[4] Centre for Health Information Computing and Statistics (CHICAS), Lancaster University, Lancaster, LA1 4YG, UK.
[5] Lancaster Ecology and Epidemiology Group (LEEG), Lancaster University, Lancaster, LA1 4YG, UK.

[&] These authors jointly supervised this work.
* Correspondent author: l.sedda@lancaster.ac.uk
[#] Shared first authors.
[$] Current address: Quanticate, Hitchin, SG5 1LH, UK.

What is already known on this topic

- Movements of doctors during their training pathway are notoriously short however a statistical quantification of these movements has not been carried out before.

- Factors associated to these movements have been investigated but not quantified.

What this study adds

- By considering a representative sample of full students' training pathways we have determined the major pattern in students' movements which is less than 50 km, although much longer movements were found during the financial crisis of 2007-2008.

For peer review only - http://bmjopen.bmj.com/site/about/guidelines.xhtml

- Identified two ethnicity backgrounds (as defined by UKMED) with those related to middle-east staying closer to their family home compared to the other (white, Chinese, black African and black Caribbean).
- Additional socio-economic factors as parent's employment and area deprivation are confirmed to be significant drivers to the movements of doctors.

Strengths and limitations

I. Use of a suite of statistical tools instead of isolated tests to ascertain significant movements scale and explanatory factors;

II. first use of UKMED full data from 2002 to 2015

III. full pathway available for only a small proportion of doctors

IV. additional socio-economic explanatory factors and policy interventions will need to be considered.

Author Contributions:

DS, CM and LS conceived and designed the study. LH, DS, JR CM and LS discussed and drafted the study protocol. LH and BF screened and selected the articles. LH and BF analysed the data. LH, BF and LS interpreted the results. LH, BF and LS drafted the manuscript. All authors critically revised the manuscript, contributed to revising the manuscript and had final responsibility for the decision to submit for publication. DS, JR, CM and LS supervised the study. The corresponding author attests that all listed authors meet authorship criteria and that no others meeting the criteria have been omitted.

Conflict of interest:

None declared

For peer review only - http://bmjopen.bmj.com/site/about/guidelines.xhtml

For peer review only

For peer review only - http://bmjopen.bmj.com/site/about/guidelines.xhtml

Abstract

Background: Throughout the UK there is a maldistribution in medical care as certain areas have a higher ratio of doctors to population than others.

Aim: This study aims to understand the movements and spatial patterns of medical students throughout their training.

Methods: Data on 97,932 UK medical students between 2002 and 2015 were collected by the General Medical Council and provided by UK Medical Education Database (UKMED). The statistical analyses were applied on the individual and aggregated movements of students from family home onto specialty training and integrating different statistical methods: Leslie models, Principal Component Analysis, Gini coefficient, chi-square test, generalised linear models and ANOVA.

Results: The majority of the movements were restricted to a distance of up 50 km; although the proportion of movements changed from year to year, with longer movements during the economic crisis of 2007-2008. At individual level, ethnicity, socio-economic class of the parents and deprivation score of the family home region were found the most important factors associated to the length of the movements. When movements were aggregated at county level, it was found that factors such as gender and qualification at entry (of medical school) were statistically associated to the movements' distances.

Conclusions: Our findings show that while the future doctors do not move far from their family home or training location, this pattern is not homogeneous over time and within individual characteristics such as ethnicity, socio-economic status and deprivation. These results may contribute to the design of intervention aimed at solving the chronic problems of maldistribution and underdoctoring in UK.

For peer review only

Introduction

The UK National Health Service (NHS) with its 1.5 million employees in England (1), 160,000 in Scotland (2), 106,000 in Wales (3) and 73,000 in Northern Ireland (4) is the fifth largest employer in the world. The ratio between number of doctors (GPs and specialist) and served population is approximately 2 per 1,000 population. The process required to become a doctor or GP is lengthy and includes multiple training stages (Medical School, 5-6 years; Foundation training, 2 years; Specialty training 3-8 years) (5). The distribution of the doctors in UK is unequal despite the sustained increase in their numbers in the last years (6). However, the chronic shortage of doctors is unlikely to be solved in short term due to structural and demographic characteristics of the underdoctored locations (7). Therefore, before any intervention is taken it is essential to understand the patterns in movements of the future doctors and which factors are behind them. Previous research had provided partial answers to these questions due to limitations in the data, methods and spatial resolution (often aggregated to large geographic areas). For example, main length of movements of the future doctors has not been statistically assessed and figures remain crude and not comparable.

In a study dating back to 1997, Parkhouse et al. (8) examined the movements between family home, medical school, foundation training and specialty training onto the career location of choice based on questionnaires. They found that only 38.2% of doctors stayed within the region of their family home, while 42% of individuals stayed in the same area as their medical school. However, at that time, London had a larger number of medical schools compared to the rest of UK. Hann et al. reported that between the mid 1980s and 2003, the maldistribution of GPs in England and Wales increased (6). This inequity reduced between 1974 and 1994, although increased again in later years and by 2006 it was above the 1974 levels (9).

While previous research focused on the use of simple confusion matrix (8), the Gini coefficient (this compares the spread of medical care to the expected distribution of doctors and GPs to find how much maldistribution occurs in the country) (10), and generalized linear models (11); they were often not sufficient to disentangle the complexity of the movements of doctors which requires a combination of these and other methodologies. For this reason, to understand the spatial scales associated to doctors' movements, in addition to the traditional statistical analyses listed above, we have employed a new method, the Leslie model (usually applied in biology). We have shown that our methods enable to provide identification of the movement scales (short to long), their differences over time and the environmental, demographic and socio-economic factors associated with these scales.

Data and Methods

For peer review only - http://bmjopen.bmj.com/site/about/guidelines.xhtml

The data was provided by the General Medical Council (GMC) through the UK Medical Education Database (UKMED) (project number UKMEDP44) and included 97,932 students who started medical school between 2002 and 2015 (which accounts for 36 medical schools). This data was the full standardised data available at UKMED at that time. For each individual, the distances between each career stage (family home to medical school, medical school to foundation, foundation to specialization) and other variables were provided (Table 1). We used the term 'ethnicity' as per UKMED database, although there are on-going debates about its appropriate use (12) in particular for medical studies (13).

Six methods were used to investigate the movements of medical students across the UK: the Leslie model (known as a Leslie matrix), principal component analysis (PCA), Gini coefficient and chi-square test to investigate the movements scales and their patterns across the years; and generalized linear models (GLM) and ANOVA to evaluate the factors influencing the movements from family home to medical school (which provide the largest data) at both individual and geographically aggregated levels.

The Leslie matrix is mostly applied to biological and ecological problems (14). The method was first developed in the 1960s and is used to model population changes over time, including migration (15). It is a symmetric matrix with positive entries where matrix cells contain the number of individuals passing from a stage to another (in a sort of pyramidical spread). In this context, the stages are the education stages (Medical School, Foundation, Specialisation). The matrix has been nested with distances, therefore for each stage, the number of students moving from one stage to the next is disaggregated by distance categories (16). The lag distance applied is 25 km (0-25 km, 25-50 km, etc…). This distance was chosen in order to have a sufficient number of counts in each distance category to conduct the analyses. Only students which nationality was English, Welsh, Scottish or Northern Irish and who were educated within the UK were considered.

The Leslie matrix was analysed by employing a principal component analysis to evaluate the most important movements' distance categories among all training stages. This approach has been proposed by Usher and Williamson (16) and the resulting eigenvectors can be compared through chi-square tests to evaluate similarities and dissimilarities in the most important movements over years (17). To confirm the presence of dominant movement distance categories, the Gini coefficient has been calculated which has been used in similar researches to estimate equalities or inequalities in the students' movements (10). The Gini coefficient varies between 0 and 1 where 0 implies complete equity, and 1 complete inequity.

Finally, a logistic Poisson generalised linear model was applied on the distances covered by each individual student (outcome) based on their individual characteristics such as gender, distances, ethnicity and socio-economic status (NSSEC and Quintile Cleaned) (full list presented in Table 1). In addition, the same model was applied to number of movements from the county of family home to county of training.

For peer review only - http://bmjopen.bmj.com/site/about/guidelines.xhtml

Since the data is aggregated by county, the model included the distance between training and family home counties and the total or modal value of each explanatory variable for both the departing (family home) and arriving (training) county. Selection of the explanatory variables was carried out by univariate modelling and backward-forward selection (18). An explanatory variable was included if significantly increased the variance explained by the model (R-squared) as tested by ANOVA. The best model was chose as the one with lowest Akaike Information Criteria (AIC) (19, 20).

Table 1: List of variables used in the analyses and their definitions. Source: UKMED data dictionary.

Variable	Description
Distance	The distance from the county of family home to the county of the medical school of choice
DistanceFMtoMS	The distance from the individual's home to the medical school of choice
DistanceMStoF1	The distance from the individual's medical school to foundation training
DistancetoF1toSP	The distance from the individual's foundation training to specialty training
Gender	The gender of the individual: 0 male, 1 female
HESA Domicile County	The county the family home is in
MSCounty	The county the medical school is in
HESA	The highest qualification the student has on entry to medical school such as A Levels
NSSEC	National Statistics Socio-Economic Classification in five -point scale:<br>1 = managerial and professional occupations<br>2 = intermediate occupations<br>3 = small employers and account workers<br>4 = lower supervisory and technical occupations<br>5 = semi-routine and routine occupations
Ethnicity L1	Level 1 information of ethnicity (full list in supplementary file)
Ethnicity L2	Level 2 information of ethnicity (full list in supplementary file)
Income Support	Whether the student's home has had any income support during their time at school

For peer review only

Bursary	Whether the individual is in the UKCAT Bursary table
Quintile	Index of multiple deprivation (IMD) of the zone the individual is from where 1 = most deprived and 5 = least deprived
BME	Ethnicity coding: Black and Minority Ethnic (BME) or white
SEC	Socioeconomic classification of the student when over 21 or student's parents (same classification as for NSSEC)
Occupation 1	Socioeconomic class occupation description of parent 1
Occupation 2	Socioeconomic class occupation description of parent 2
School Type	What type of school the student attended between the ages of 11 and 16

156

For peer review only

For peer review only - http://bmjopen.bmj.com/site/about/guidelines.xhtml

Results

Of 97,932 students only for 871 is available the full path from family home to specialisation. The 871 students registered between 2002 and 2009 are representative of the full dataset as shown in Supplementary Figures A1, A2 and A3. The analyses included 36 medical schools and all the hospitals providing training to the doctors across the UK. The first four analyses (Leslie, Gini, PCA and chi-square test) were applied to the 871 records, while the Poisson GLM and ANOVA on the whole 97,932 records.

The Leslie matrix, applied to only the students for which the full pathway is known, found that out of 40 principal components (one for each distance category), the first principal component (distances up to 25 km) explained 41% of the variance in the general movements pattern, the second 13% (25-50 km) and the third 8% (50-75 km) (Supplementary Table A1). The highest proportion of students (54%) concentrates in the shorter movement distances (up to 50 km) than longer movement distances. These results have been confirmed by the value of the Gini Coefficient for the Leslie matrix, which was 0.936 (Figure 1) meaning inequity on the distribution of movements among all categories. These results indicate that most of the students decided to stay near their home, medical school, foundation or specialty training region.

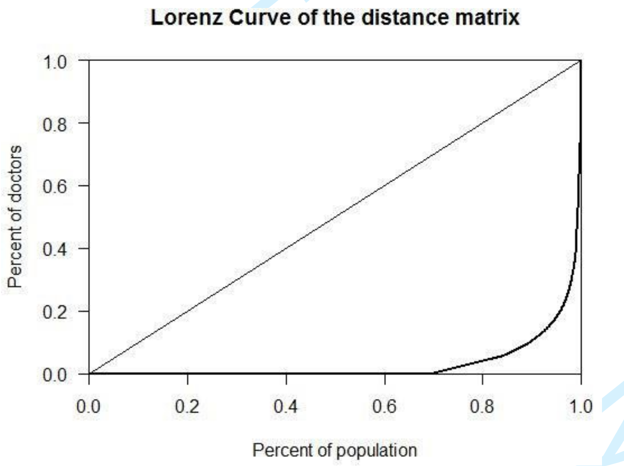

Figure 1: Lorenz curve of the Leslie Matrix. Equality (Gini coefficient equal to 0) is achieved when the Lorenz curve (bold black line) coincide with the diagonal. The diagonal represents the status where all distances have equal number of students. In this figure a strong inequality is shown, with the entire Lorenz curve laying on values above 0.7.

The proportions of movements among the distance categories are heterogeneous in time. These differences have been tested by chi-square test applied to paired first principal components of annual Leslie matrices (from 2002 to 2009 – where 2009 is the last year for which we have a full pathway for

For peer review only - http://bmjopen.bmj.com/site/about/guidelines.xhtml

students). Figure 2 shows that 2002 proportion of short movements are significantly different from most of the rest of the years, apart from 2007. 2004 was similar only to 2003 and 2009; 2006 to 2005, 2007 and 2009; and 2008 to 2005, 2007 and 2009. The year that was similar to most of all the other years is 2009, which was different only to 2002 (Figure 2). Notably, the years with longest distances where 2007 and 2008 where the majority of students travelled distances up to 100-125 km (Supplementary Table A1).

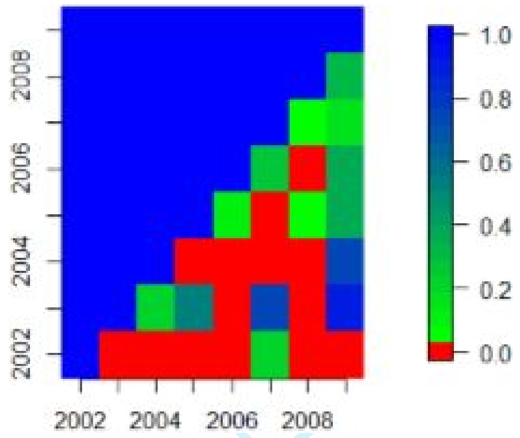

Figure 2: Chi-square *p-values* from comparison of annual first components of the Leslie matrix. *p-values* below 0.05 are highlighted red.

To evaluate determinants of the movement from family home to medical school at both individual and aggregated (by county) level, generalised linear models were employed within a variable selection frameworks, the latter aimed at the largest variance explained by each variable (ANOVA) and lowest AIC for the model fitting. These analyses were restricted to the family home to medical school data only because including all the records in the dataset.

At individual level, the best model fitting of the distances travelled by students was obtained with ethnicity sub-group (Level 2 information), NSSEC and IMD (Quintile) (Supplementary Table A2). All the classes of deprivation and NSSEC were found statistically significant. In terms of deprivation, compared to the most deprived, the least deprived levels (3 to 5) travelled a shorter distance when they moved from their family home to medical school. Compared to students with parents in managerial and professional occupation, the students with other occupations travelled shorter distances (see NSSEC levels in Supplementary Table A2). In terms of the ethnicity variable (Supplementary file A for full list), it was possible to distinguish two groups: those travelling lower distances: Arab, Bangladeshi, Indian, Pakistani, ‘other Asian background’ ‘other mixed background’ and ‘other ethnic group’; from those travelling longer distances: British, Chinese, Gypsy or Irish traveler, Irish, Scottish, White and Asian, White and Black Caribbean, White and black African, and ‘other white background’. A three-fold

For peer review only - http://bmjopen.bmj.com/site/about/guidelines.xhtml

difference was found between British and African in their longer movements (with British moving further), while a 1.6-fold difference was found between Arab and Indian in their shorter movements (with Arab staying closer).

The best fitted model by county confirms the importance of IMD in the number of students movements in both arriving and departing counties. Distance between counties was an important factor for the number of students moving from one county to the other, with medical schools having more students from neighbouring counties than for counties far apart. The rest of the most significant variables were all associated to the departing county: BME, total number of female students, highest qualification at entry, and SEC (Supplementary Table A3). In particular, counties with higher proportions of BME or with higher deprivation or socio-economic status were associated with less movements. While, counties with a larger proportion of female students or higher qualification at entry were associated with larger number of movements.

Discussion

This research found that the largest proportion of students travelled within 25 km in their various stages of professional development, with most of the movements been statistically significantly at less than 50 km, although not every year. In fact, in the years of the financial crisis (2007-2008) (21) students travelled further (up to 125 km). No previous studies assessed the effect of financial crisis on students' movements in UK, however a study on Spanish students reported that students focused more on career perspectives than lifestyle in response of the economic crisis (22). If this was the reason for longer movements by UK students it is something that will need to be ascertain.

The up-to 50 km modal distance for all the rest of the years is the first statistical quantification of UK-borne students in scientific literature. A GMC report that focused on doctors graduating between 2012 and 2018 found that almost a quarter (23%) stayed within 10 miles (16 km) of where they studied at medical school (23). Despite the absence of distance information, a previous study (on a restricted number of doctors and obtained by questionnaire) reported that between 1974 and 2008, 36% of the doctors stayed in the same region as their family home during medical school and 48% undertook specialist training in the same region as their medical school (24). A more recent observational study that focused on doctors graduating between 2012 and 2014, found that the majority of doctors decide to train in foundation schools that are close to their family homes (25).

We found strong inequity in the movements – e.g. the presence of dominant distance categories compared to others. A previous study using Gini coefficient applied to movements for general practitioners between 1974 and 2003 (6) found more equality (Gini coefficient between 0.4 and 0.5) than

For peer review only - http://bmjopen.bmj.com/site/about/guidelines.xhtml

the present research. A direct comparison between the two studies cannot be done, but the increase in supply since 1974 (6) could have caused a more pronounced movements inequality (as we found) than previously.

The main factors associated to students' movement distances and student counts from family home to medical school are very similar at both individual and aggregated – by county – levels. The most important factors were socioeconomic status, deprivation score (IMD Quintile) and ethnicity (BME, Ethnicity group and sub-groups). In our analyses, increased deprivation showed to be associated to both a lower number of doctors moving and shorter distances moved, while students with higher degrees at entry in the medical school or parents with professional occupation tend to move further away. In previous literature, Primary Care Trusts (PCT) in England are seen to be attractive if there is less crime in the area, or better schools and opportunities (9). Similarly those students with parents in NSSEC1 (professional occupations) were less likely to work in deprived areas compared to those in NSSEC5 (routine occupations) (26). Regarding the ethnicity, one study reported that doctors from non-white ethnic backgrounds were more likely to choose to work in a foundation trust closer to their family home (24). We found the same results though not all the non-white ethnic groups are staying closer. In fact, we were able to distinguish the group moving closer to their family home, mainly from middle east and Indian background from those that moved further (the rest of the groups), although it is important to underline the geographical distribution of ethnic minority in UK which is clustered (27). Personal factors such as ethnicity, education and gender were all significant contributing factors to the movement of doctors at both individual and county levels (28).

Considering structural elements of our societies, such as deprivation, and demographic ones such as ethnicity, will require different types, spatial scales and economic effort for improving the distribution of doctors in UK. NHS proposed a 'rural weighting' payment which would aim to attract doctors and GPs to rural areas with a financial incentive (29). This is equally applicable in areas of high deprivation. Payments of this type could also be increased in line with the length of service thereby increasing retention of medical practitioners within rural or deprived areas. Another broader solution to the problem is widening access to medical care as it has been found that there is a link between childhood background and the class of population the GPs are likely to serve (26).

The present study did not consider international medical graduates who work in UK, or UK-borne doctors working abroad; neither the doctors movements from when the records began. A new study (NIHR134540) (30) is investigating why doctors work where they work and how this is influenced by the organisation of the healthcare system, the social and physical environment where the future doctors grow up and move as well as their individual belief and choices. This study which is based on mixed methods and a larger dataset than any previously used will be able to increase the understanding around doctors

For peer review only

For peer review only - http://bmjopen.bmj.com/site/about/guidelines.xhtml

movements from the doctor's perspectives as well and not only from data records. We hope that our study and the ones currently active will support the best interventions to solve the underdoctoring and maldistribution of doctors in the UK.

Data source

UK Medical Education Database ("UKMED") UKMEDP044 extract generated on 01/06/2018. Approved for publication on 30/06/2023.

Acknowledgments

I am grateful to UKMED for the use of these data. However, UKMED bears no responsibility for their analysis or interpretation. The data includes information derived from that collected by the Higher Education Statistics Agency Limited ("HESA") and provided to the GMC ("HESA Data"). Source: HESA Student Records 2002/2015 Copyright Higher Education Statistics Agency Limited. The Higher Education Statistics Agency Limited makes no warranty as to the accuracy of the HESA Data, cannot accept responsibility for any inferences or conclusions derived by third parties from data or other information supplied by it.

Patient and Public involvement

Patients or the public were not involved in the design, or conduct, or reporting, or dissemination plans of our research.

References

1. Trust N. The NHS workforce in numbers 2022 [Available from: https://www.nuffieldtrust.org.uk/resource/the-nhs-workforce-in-numbers.
2. Government S. Health Workforce [Available from: https://www.gov.scot/policies/health-workforce/.
3. Government W. Staff directly employed by the NHS: as at 30 September 2022 2022 [Available from: https://www.gov.wales/staff-directly-employed-nhs-30-september-2022-html.
4. Health Do. Northern Ireland health and social care (HSC) workforce statistics December 2022 2023 [Available from: https://www.health-ni.gov.uk/publications/northern-ireland-health-and-social-care-hsc-workforce-statistics-december-2022.
5. Brice J, Corrigan O. The changing landscape of medical education in the uk. . Medical Teacher. 2010;32(9):727-32.
6. Hann M, Gravelle H. The maldistribution of general practitioners in england and wales: 1974-2003. The British journal of general practice : the journal of the Royal College of General Practitioners. 2004;54(509).

For peer review only - http://bmjopen.bmj.com/site/about/guidelines.xhtml

7. British Medical Association. NHS medical staffing data analysis2023 06/07/2023. Available from: https://www.bma.org.uk/advice-and-support/nhs-delivery-and-workforce/workforce/nhs-medical-staffing-data-analysis.
8. Parkhouse J, Lambert T. Home, training and work: Mobility of british doctors. 1997;31:399-407.
9. Goddard M, Gravelle H, Hole A, Marini G. Where did all the gps go? increasing supply and geographical equity in england and scotland. Journal of health services research & policy,. 2010;15(1):28-35.
10. Morita T, Tanimoto T, Morita M, Tsubokura M, M. K. Trend in unequal geo-graphical distribution of doctors by age and sex in japan from 2004 to 2014. Public Health. 2018;159:95-8.
11. Ide H, Doi S, Atarashi H, Fujita S, Koike S. The distance and chance of lifetime geographical movement of physicians in japan: an analysis using the age-period-cohort model. Human Resources for Health. 2018;16(1).
12. Flanagin A, Frey T, Christiansen SL. Updated Guidance on the Reporting of Race and Ethnicity in Medical and Science Journals. Jama-J Am Med Assoc. 2021;326(7):621-7.
13. Martinez RAM, Andrabi N, Goodwin AN, Wilbur RE, Smith NR, Zivich PN. Conceptualization, Operationalization, and Utilization of Race and Ethnicity in Major Epidemiology Journals, 1995-2018: A Systematic Review. Am J Epidemiol. 2023;192(3):483-96.
14. Monte L. Characterisation of a nonlinear leslie matrix model for predicting the dy- namics of biological populations in polluted environments: Applications to radioecology. Ecological Modelling. 2013;248:174-83.
15. Leslie P. On the use of matrices in certain population mathematics. 1945;33:183-212.
16. Usher M, Williamson M. A deterministic matrix model for handling the birth, death, and migration processes of spatially distributed populations. . 1970;26:1-12.
17. Freund R. Symmetric matrices and eigendecomposition2014. Available from: http://s3.amazonaws.com/mitsloan-php/wp-faculty/sites/30/2016/12/15032137/Symmetric-Matrices-and-Eigendecomposition.pdf.
18. Upton G, Cook I. Forward selection 2014 [Available from: http://www.oxfordreference.com/view/10.1093/acref/9780199679188.001.0001/acref-9780199679188-e-624.
19. Symonds M, Moussalli A. A brief guide to model selection, multimodel inference and model averaging in behavioural ecology using akaike's information criterion. Behavioral Ecology and Sociobiology. 2011;65(1):13-21.
20. Gelman A. Discussion paper: Analysis of variance - why it is more important than ever. The Annals of Statistics. 2005;33(1):1-33.
21. Lindstrom M, Giordano GN. The 2008 financial crisis: Changes in social capital and its association with psychological wellbeing in the United Kingdom - A panel study. Soc Sci Med. 2016;153:71-80.
22. Harris JE, Lopez-Valcarcel BG, Ortun V, Barber P. Specialty choice in times of economic crisis: a cross-sectional survey of Spanish medical students. Bmj Open. 2013;3(2).
23. GMC. The state of medical education and practice in the UK 2019 [Available from: https://www.gmc-uk.org/-/media/documents/the-state-of-medical-education-

For peer review only - http://bmjopen.bmj.com/site/about/guidelines.xhtml

and-practice-in-the-uk---workforce-report_pdf-80449007.pdf.
24. Goldacre M, Davidson J, Maisonneuve J, Lambert T. Geographical movement of doctors from education to training and eventual career post: UK cohort studies. J R Soc Med. 2013;106(3):96-104.
25. Kumwenda B, Cleland JA, Prescott GJ, Walker KA, Johnston PW. Geographical mobility of UK trainee doctors, from family home to first job: a national cohort study. BMC Med Educ. 2018;18(1):314.
26. Dowell J, Norbury M, Steven K, Guthrie B. Widening access to medicine may improve general practitioner recruitment in deprived and rural communities: survey of gp origins and current place of work. BMC Medical Education. 2015;15(1).
27. Shaw B, Menzies L, Bernardes E, Baars S, Nye P, Allen R. Ethnicity, gender and social mobility. London: Social Mobility Commission. 2016.
28. Dussault G, Franceschini M. Not enough there, too many here: understanding geographical imbalances in the distribution of the health workforce. Human Resources for Health. 2006.
29. RSN RSN. Shortage of rural doctors puts patients' health at risk. 2017 [Available from: http://rsnonline.org.uk/shortage-of-rural-doctors-puts-patients-health-at-risk.
30. NIHR. HSDR Funding Committee Public Minutes March 2022 2022 [Available from: https://www.nihr.ac.uk/documents/hsdr-funding-committee-public-minutes-march-2022/30467.

For peer review only - http://bmjopen.bmj.com/site/about/guidelines.xhtml

Supplementary File

Supplementary information A:

Level 1 (bold) and Level 2 Ethnicity.
https://www.ethnicity-facts-figures.service.gov.uk/style-guide/ethnic-groups

Asian or Asian British

- Indian
- Pakistani
- Bangladeshi
- Chinese
- Any other Asian background

Black, African, Caribbean or Black British

- African
- Caribbean
- Any other Black, African or Caribbean background

Mixed or multiple ethnic groups

- White and Black Caribbean
- White and Black African
- White and Asian
- Any other Mixed or multiple ethnic background

White

- English, Welsh, Scottish, Northern Irish or British
- Irish
- Gypsy or Irish Traveller
- Any other White background

Other ethnic group

- Arab
- Any other ethnic group

Supplementary figures

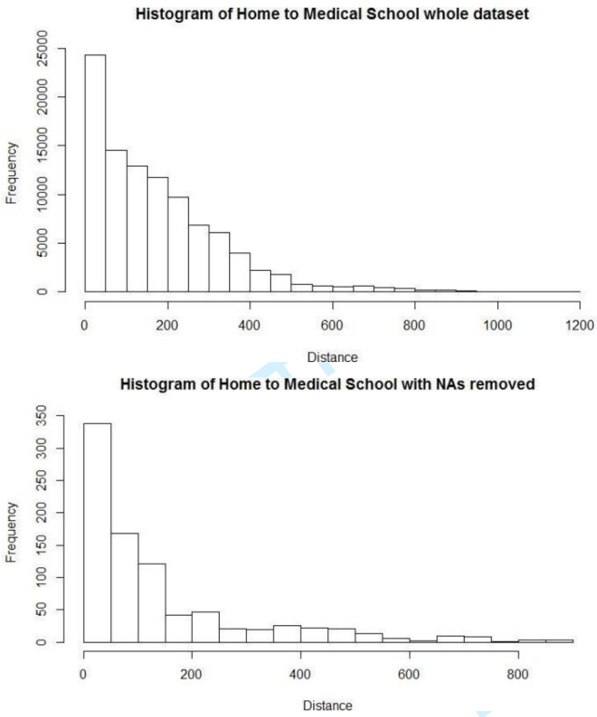

Figure A1: Histograms of the full records (up) and 871 records with full pathway (down) of the distances from family home to medical school.

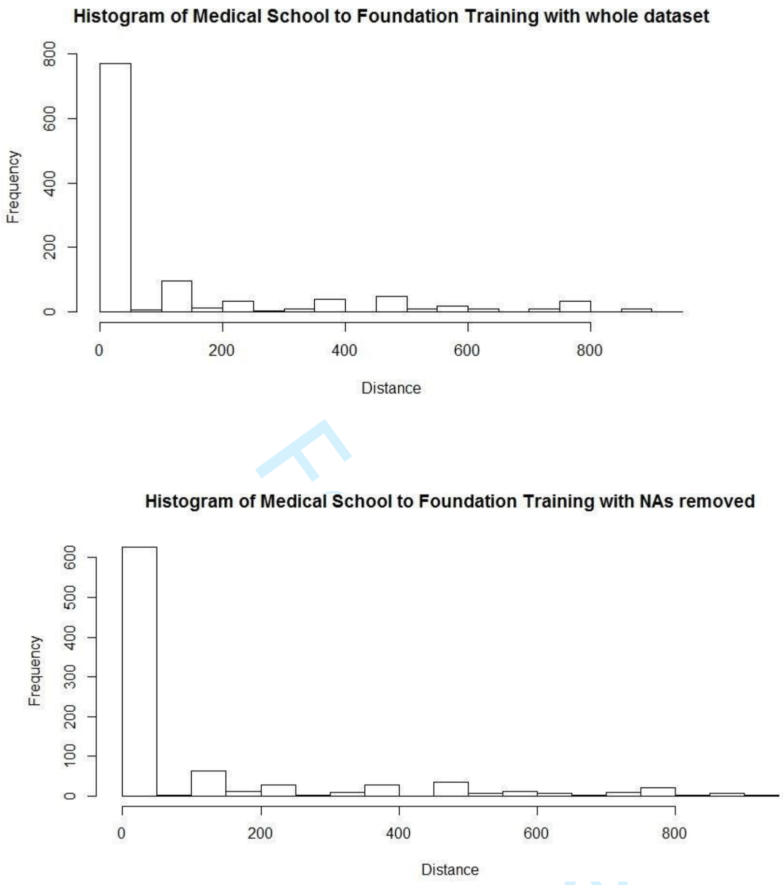

Figure A2: Histograms of the full records (up) and 871 records with full pathway (down) of the distances from medical school to foundation training.

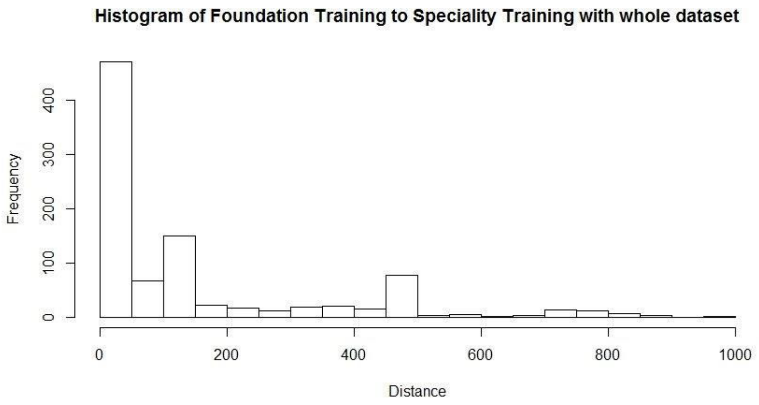

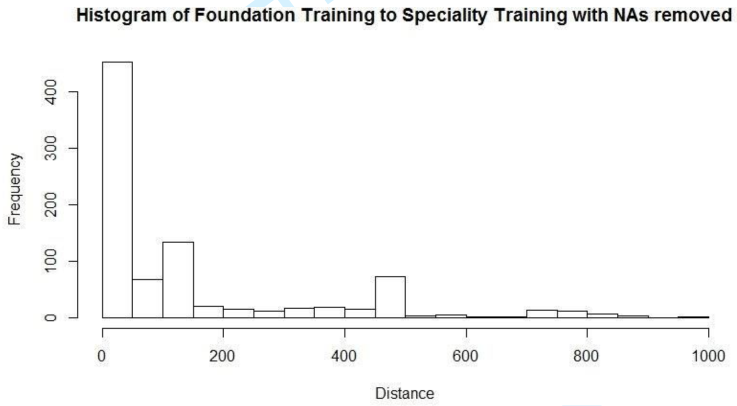

Figure A3: Histograms of the full records (up) and 871 records with full pathway (down) of the distances from foundation training to speciality.

For peer review only - http://bmjopen.bmj.com/site/about/guidelines.xhtml

Supplementary Tables

Table A1: Proportion of variance explained by the distance categories for all years and by each year from principal component analysis applied to all years and yearly Leslie matrices respectively.

km	Overall	2002	2003	2004	2005	2006	2007	2008	2009
0-25	40.98%	42.70%	40.07%	43.12%	45.34%	31.28%	28.55%	28.19%	43.45%
25-50	13.19%	1.32%	12.17%	15.84%	15.87%	10.82%	5.58%	4.72%	14.91%
50-75	7.84%	5.27%	6.47%	9.07%	9.83%	2.62%	6.01%	0%	0%
75-100	4.75%	5.09%	4.98%	5.25%	2.94%	8.12%	7.48%	6.17%	0%
100-125	8.47%	11.15%	7.34%	7.73%	8.36%	6.91%	12.70%	21.80%	0%
125-150	6.00%	9.83%	7.06%	3.07%	3.78%	15.86%	14.69%	7.72%	10.68%
150-175	1.11%	1.50%	1.29%	0.45%	1.23%	4.31%	1.11%	<0.1%	0%
175-200	1.35%	2.39%	1.61%	1.24%	0.37%	0.76%	2.53%	1.54%	6.69%
200-225	0.74%	0%	0.72%	0.57%	0.28%	2.01%	0.49%	4.64%	0%
225-250	1.17%	5.95%	2.06%	1.62%	0.64%	1.62%	0%	1.46%	4.59%
250-275	0.51%	2.26%	0.77%	0.21%	0.23%	0.51%	1.11%	1.13%	1.81%
275-300	0.36%	0%	0.72%	<0.1%	0.59%	0%	0%	0%	0%
300-325	1.34%	0.17%	3.97%	0.22%	0.63%	0.14%	1.15%	0.23%	0%
325-350	0.42%	1.20%	0.46%	0.40%	0.22%	<0.1%	1.11%	0.15%	0%
350-375	1.50%	1.36%	2.26%	1.69%	0.61%	3.88%	0%	3.08%	0%
375-400	0.78%	0%	1.24%	1.15%	0.43%	0%	2.18%	<0.1%	0%
400-425	0.56%	0%	1.01%	0.39%	0.41%	2.19%	<0.1%	1.07%	0%
425-450	0.58%	0%	0.44%	0.58%	0.78%	2.16%	0%	<0.1%	0%
450-475	4.43%	4.60%	2.32%	5.07%	4.20%	2.42%	5.66%	1.93%	0%
475-500	0.36%	1.19%	0.51%	0.26%	0.30%	1.01%	0%	1.48%	0%
500-525	0.33%	1.65%	0.27%	<0.1%	0.20%	0%	0%	1.47%	0%
525-550	<0.1%	0%	<0.1%	<0.1%	<0.1%	0.57%	1.07%	<0.1%	0%
550-575	0.27%	0%	0%	0%	0.20%	0.41%	4.04%	0.68%	0%
575-600	0.22%	0%	0.43%	<0.1%	0.14%	0.99%	0%	0%	0%
600-625	0.14%	0%	0%	0.21%	0.28%	0%	0%	0%	0%
625-650	<0.1%	0%	0%	0.18%	<0.1%	0%	1.08%	0%	0%

650-675	0.16%	0%	0.21%	<0.1%	0.20%	<0.1%	1.07%	0%	0%
675-700	0.11%	<0.1%	0%	<0.1%	0.20%	0%	0%	0%	7.90%
700-725	0.26%	1.13%	<0.1%	<0.1%	0.26%	0%	0.99%	1.39%	0%
725-750	0.42%	0%	0.65%	0.23%	0.41%	0%	1.10%	6.30%	0%
750-775	0.18%	0%	<0.1%	<0.1%	0.58%	<0.1%	0%	<0.1%	0%
775-800	0.71%	1.20%	0.44%	0.97%	<0.1%	<0.1%	0.23%	2.46%	0%
800-825	0.13%	0%	0%	0.19%	0.26%	0%	<0.1%	0%	0%
825-850	0.17%	0%	0.24%	0.18%	<0.1%	0%	0%	0%	0%
850-875	0.18%	0%	0%	0%	0.20%	0%	<0.1%	0%	9.97%
875-900	<0.1%	0%	<0.1%	0%	0%	1.34%	0%	2.15%	0%
900-925	<0.1%	<0.1%	0%	0%	0%	0%	0%	0%	0%
925-950	0%	0%	0%	0%	0%	0%	0%	0%	0%
950-975	<0.1%	0%	0.20%	0%	0%	0%	0%	0%	0%

For peer review only

Table A2: Generalised liner model. Best fitted model for individual data: statistical summary of the explanatory variables' coefficients. REF: reference category.

Variable	Estimate	Standard Error	*p-value*
Intercept	5.1971	0.0147	<2x10-16
NSSEC (Level 1)	REF		
NSSEC (Level 2)	-0.0865	0.0018	<2x10-16
NSSEC (Level 3)	-0.0789	0.0017	<2x10-16
NSSEC (Level 4)	-0.1112	0.0032	<2x10-16
NSSEC (Level 5)	-0.1207	0.0029	<2x10-16
Quintile (Level 1)	REF		
Quintile (Level 2)	0.0108	0.0009	<2x10-16
Quintile (Level 3)	-0.0357	0.0011	<2x10-16
Quintile (Level 4)	-0.0695	0.0012	<2x10-16
Quintile (Level 5)	-0.2106	0.0018	<2x10-16
African	0.0356	0.0149	0.0165
Arab	-0.2447	0.0154	<2x10-16
Bangladeshi	-0.2616	0.0154	<2x10-16
British	0.1009	0.0147	6.32x10-12
Caribbean	-0.0157	0.0159	0.3250
Chinese	0.0634	0.0149	2.07x10-5
Gypsy or Irish Traveler	0.1800	0.0423	2.13x10-5
Indian	-0.1570	0.0147	<2x10-16
Irish	0.3473	0.0148	<2x10-16
Pakistani	-0.2225	0.0148	<2x10-16
White and Asian	0.0749	0.0149	4.66x10-7
White and Black African	0.1340	0.0159	<2x10-16
White and Black Caribbean	0.0920	0.0158	5.55x10-9
Other Asian background	-0.1969	0.0148	<2x10-16
Other Black background	0.0355	0.0209	0.0892

For peer review only

Other Mixed background	-0.0461	0.0150	0.0022
Other White background	0.1077	0.0147	2.23x10-13
Other Ethnic group	-0.1702	0.0151	<2x10-16
Not stated	0.0814	0.0223	0.0003

For peer review only

Table A3: Generalised linear model. Best fitted model for counts of movements by county: statistical summary of the explanatory variables' coefficients. Depart = county of family home; Arrive = county with the Medical School.

Variable	Estimate	Standard Error	*p-value*
Intercept	-9.629x10-01	5.862x10-02	<2x10-16
Distance	-5.998x10-03	3.175x10-05	<2x10-16
BME Depart	-1.204	4.257x10-02	<2x10-16
Gender Depart	2.691	2.909x10-02	<2x10-16
HESA Depart	8.470x10-02	3.754x10-03	<2x10-16
Quintile Depart	-4.540x10-01	4.886x10-03	<2x10-16
Quintile Arrive	-4.833x10-01	6.155x10-03	<2x10-16
SEC Depart	-1.834x10-01	2.093x10-02	<2x10-16

For peer review only

Reporting checklist for cohort study.

Based on the STROBE cohort guidelines.

Instructions to authors

Complete this checklist by entering the page numbers from your manuscript where readers will find each of the items listed below.

Your article may not currently address all the items on the checklist. Please modify your text to include the missing information. If you are certain that an item does not apply, please write "n/a" and provide a short explanation.

Upload your completed checklist as an extra file when you submit to a journal.

In your methods section, say that you used the STROBE cohortreporting guidelines, and cite them as:

von Elm E, Altman DG, Egger M, Pocock SJ, Gotzsche PC, Vandenbroucke JP. The Strengthening the Reporting of Observational Studies in Epidemiology (STROBE) Statement: guidelines for reporting observational studies.

		Reporting Item	Page Number
**Title and abstract**			
Title	#1a	Indicate the study's design with a commonly used term in the title or the abstract	1
Abstract	#1b	Provide in the abstract an informative and balanced summary of what was done and what was found	3
**Introduction**			
Background / rationale	#2	Explain the scientific background and rationale for the investigation being reported	4
Objectives	#3	State specific objectives, including any prespecified hypotheses	4
**Methods**			
Study design	#4	Present key elements of study design early in the paper	5
Setting	#5	Describe the setting, locations, and relevant dates, including periods of recruitment, exposure, follow-up, and data	5

For peer review only

		collection	
Eligibility criteria	#6a	Give the eligibility criteria, and the sources and methods of selection of participants. Describe methods of follow-up.	n/a no selection
Eligibility criteria	#6b	For matched studies, give matching criteria and number of exposed and unexposed	n/a no matched study
Variables	#7	Clearly define all outcomes, exposures, predictors, potential confounders, and effect modifiers. Give diagnostic criteria, if applicable	6 and 7
Data sources / measurement	#8	For each variable of interest give sources of data and details of methods of assessment (measurement). Describe comparability of assessment methods if there is more than one group. Give information separately for for exposed and unexposed groups if applicable.	5
Bias	#9	Describe any efforts to address potential sources of bias	2
Study size	#10	Explain how the study size was arrived at	n/a all database considered
Quantitative variables	#11	Explain how quantitative variables were handled in the analyses. If applicable, describe which groupings were chosen, and why	5 and 6
Statistical methods	#12a	Describe all statistical methods, including those used to control for confounding	
5 and 6			
Statistical methods	#12b	Describe any methods used to examine subgroups and interactions	5 and 6
Statistical methods	#12c	Explain how missing data were addressed	n/a no missing data
Statistical methods	#12d	If applicable, explain how loss to follow-up was addressed	n/a
Statistical methods	#12e	Describe any sensitivity analyses	
n/a Different models were tested (supplementary)			

For peer review only

Results

Participants	#13a	Report numbers of individuals at each stage of study—eg numbers potentially eligible, examined for eligibility, confirmed eligible, included in the study, completing follow-up, and analysed. Give information separately for for exposed and unexposed groups if applicable.	n/a
Participants	#13b	Give reasons for non-participation at each stage	n/a
Participants	#13c	Consider use of a flow diagram	
n/a			
Descriptive data	#14a	Give characteristics of study participants (eg demographic, clinical, social) and information on exposures and potential confounders. Give information separately for exposed and unexposed groups if applicable.	n/a
Descriptive data	#14b	Indicate number of participants with missing data for each variable of interest	
n/a			
Descriptive data	#14c	Summarise follow-up time (eg, average and total amount)	
n/a			
Outcome data	#15	Report numbers of outcome events or summary measures over time. Give information separately for exposed and unexposed groups if applicable.	
9			
Main results	#16a	Give unadjusted estimates and, if applicable, confounder-adjusted estimates and their precision (eg, 95% confidence interval). Make clear which confounders were adjusted for and why they were included	n/a Supplementary file
Main results	#16b	Report category boundaries when continuous variables were categorized	n/a
Main results	#16c	If relevant, consider translating estimates of relative risk into absolute risk for a meaningful time period	
n/a			

For peer review only

Other analyses	#17	Report other analyses done—eg analyses of subgroups and interactions, and sensitivity analyses	n/a Supplementary file
**Discussion**			
Key results	#18	Summarise key results with reference to study objectives	10 and 11
Limitations	#19	Discuss limitations of the study, taking into account sources of potential bias or imprecision. Discuss both direction and magnitude of any potential bias.	2
Interpretation	#20	Give a cautious overall interpretation considering objectives, limitations, multiplicity of analyses, results from similar studies, and other relevant evidence.	10 and 11
Generalisability	#21	Discuss the generalisability (external validity) of the study results	10 and 11
**Other Information**			
Funding	#22	Give the source of funding and the role of the funders for the present study and, if applicable, for the original study on which the present article is based	12

Notes:

- 6a: n/a no selection
- 6b: n/a no matched study
- 10: n/a all database considered
- 12c: n/a no missing data
- 12e: n/a Different models were tested (supplementary)
- 16a: n/a Supplementary file
- 17: n/a Supplementary file The STROBE checklist is distributed under the terms of the Creative Commons Attribution License CC-BY. This checklist was completed on 06. July 2023 using https://www.goodreports.org/, a tool made by the EQUATOR Network in collaboration with Penelope.ai

For peer review only

BMJ Open

Determining the distance patterns in the movements of future doctors in UK between 2002 and 2015: a retrospective cohort study

Journal:	*BMJ Open*
Manuscript ID	bmjopen-2023-077635.R1
Article Type:	Original research
Date Submitted by the Author:	15-Jan-2024

Complete List of Authors:	Hitchings, Lucy; Lancaster University<br>Fleet, Ben; Lancaster University, Medical School<br>Smith, Daniel; General Medical Council<br>Read, Jonathan; Lancaster University Faculty of Health and Medicine, Centre for Health Informatics, Computing and Statistics, Lancaster Medical School<br>Melville, Colin; The University of Manchester; General Medical Council Manchester<br>Sedda , Luigi ; Lancaster University

**Primary Subject Heading**:	Medical education and training
Secondary Subject Heading:	Health policy, Medical management
Keywords:	HEALTH SERVICES ADMINISTRATION & MANAGEMENT, EDUCATION & TRAINING (see Medical Education & Training), STATISTICS & RESEARCH METHODS

SCHOLARONE™
Manuscripts

I, the Submitting Author has the right to grant and does grant on behalf of all authors of the Work (as defined in the below author licence), an exclusive licence and/or a non-exclusive licence for contributions from authors who are: i) UK Crown employees; ii) where BMJ has agreed a CC-BY licence shall apply, and/or iii) in accordance with the terms applicable for US Federal Government officers or employees acting as part of their official duties; on a worldwide, perpetual, irrevocable, royalty-free basis to BMJ Publishing Group Ltd ("BMJ") its licensees and where the relevant Journal is co-owned by BMJ to the co-owners of the Journal, to publish the Work in this journal and any other BMJ products and to exploit all rights, as set out in our licence.

The Submitting Author accepts and understands that any supply made under these terms is made by BMJ to the Submitting Author unless you are acting as an employee on behalf of your employer or a postgraduate student of an affiliated institution which is paying any applicable article publishing charge ("APC") for Open Access articles. Where the Submitting Author wishes to make the Work available on an Open Access basis (and intends to pay the relevant APC), the terms of reuse of such Open Access shall be governed by a Creative Commons licence – details of these licences and which Creative Commons licence will apply to this Work are set out in our licence referred to above.

Other than as permitted in any relevant BMJ Author's Self Archiving Policies, I confirm this Work has not been accepted for publication elsewhere, is not being considered for publication elsewhere and does not duplicate material already published. I confirm all authors consent to publication of this Work and authorise the granting of this licence.

For peer review only

Determining the distance patterns in the movements of future doctors in UK between 2002 and 2015: a retrospective cohort study

Lucy A. Hitchings[1,#], Benjamin G. Fleet[2,#], Daniel Smith[3,&], Jonathan Read[4,&], Colin Melville[3,5,&], Luigi Sedda[6,&,*]

[1] Mathematics and Statistics, Lancaster University, Lancaster, LA1 4YG, UK.

[2] Lancaster Medical School, Lancaster University, Lancaster, LA1 4YG, UK.

[3] General Medical Council, Manchester, M3 3AW, UK.

[4] Centre for Health Information Computing and Statistics (CHICAS), Lancaster University, Lancaster, LA1 4YG, UK.

[5] School of Medical Sciences, Manchester University, M13 9PL, UK

[6] Lancaster Ecology and Epidemiology Group (LEEG), Lancaster University, Lancaster, LA1 4YG, UK.

[&] These authors jointly supervised this work.

* Correspondent author: l.sedda@lancaster.ac.uk

[#] Shared first authors.

For peer review only

For peer review only - http://bmjopen.bmj.com/site/about/guidelines.xhtml

Abstract

Objective To determine and identify distance patterns in the movements of medical students and junior doctors between their training locations.

Design A retrospective cohort study of UK medical students from 2002 to 2015.

Setting All UK medical schools, foundation and specialty training organisation.

Participants All UK medical students from 2002 to 2015, for a total of 97,932 participants.

Outcome measures Individual movements and number of movements by county of students from family home to medical school training, from medical school to foundation training and from foundation to specialty training.

Methods Leslie matrix, Principal Component Analysis, Gini coefficient, chi-square test, generalised linear models and variable selection methods were employed to explore the different facets of students and junior doctors' movements from family home to medical school and for the full pathway.

Results The majority of the movements between the different training stages were restricted to a distance of up to 50 km; although the proportion of movements changed from year to year, with longer movements during 2007-2008. At the individual level, ethnicity, socio-economic class of the parent(s), and the deprivation score of the family home region were found to be the most important factors associated with the length of the movements from family home to medical school. Similar results were found when movements were aggregated at the county level, with the addition of factors such as gender and qualification at entry (to medical school) being statistically associated with the number of students moving between counties.

Conclusion Our findings show that while the future doctors do not move far from their family home or training location, this pattern is not homogeneous over time. Distances are influenced by demographic, socio-economic status and deprivation. These results may contribute in designing interventions aimed at solving the chronic problems of maldistribution and underdoctoring in UK.

Strengths and limitations of this study

For peer review only - http://bmjopen.bmj.com/site/about/guidelines.xhtml

- Use of a suite of statistical tools instead of isolated tests to ascertain significant movements scale and explanatory factors;
- first use of UKMED full data from 2002 to 2015
- full pathway available for only a small proportion of doctors
- additional socio-economic explanatory factors and policy interventions will need to be considered.

For peer review only

Introduction

The UK National Health Service (NHS) with its 1.5 million employees in England (1), 160,000 in Scotland (2), 106,000 in Wales (3), and with the Health and Social Care 73,000 employees in Northern Ireland (4), is the fifth largest employer in the world (5). The ratio between number of doctors (GPs and specialist) and served population is approximately 2 per 1,000 population. The process required to become a doctor or GP is lengthy and includes multiple training stages: Medical School, (4-6 years); Foundation training (2 years); and Specialty training (3-8 years) (6). The distribution of the doctors to population served in the UK is unequal (as shown for GPs in the past (7)), despite the sustained increase in doctor numbers in the last few years (1). However, the shortage of doctors is unlikely to be solved in the short term due to the structural and demographic characteristics of the underdoctored locations (8). Therefore, before any intervention is taken it is essential to understand the patterns in geographical movements of doctors and which factors are behind them. In fact, the potential maldistribution of doctors in the United Kingdom is contingent upon the spatial mobility of medical professionals, specifically in terms of their divergence from their geographic origins or training locations. If doctors predominantly remain within close proximity to their hometowns or training institutions, the resultant geographic distribution of healthcare providers is likely to mirror the demographic characteristics of medical school entrants and the geographic locales of these educational institutions and postgraduate training facilities. This, in turn, could deviate from an ideal distribution aligned with the broader healthcare needs of the population.

Previous research had provided partial answers to the questions regarding the patterns of movements of future doctors due to limitations in the data, methods and coarse spatial resolution. So far, analyses of these patterns has been descriptive and hardly statistically assessed, therefore figures remain crude and not comparable.

In a study dating back to 1997, Parkhouse et al. (9) found that only 38.2% of doctors stayed within the region of their family home, while 42% of individuals stayed in the same area as their medical school. However, at that time, London had a larger number of medical schools compared to the rest of UK. Hann et al. reported that between the mid 1980s and 2003, the maldistribution of GPs in England and Wales increased (7). This reduced between 1974 and 1994, although increased again in later years and by 2006 it was greater than the 1974 levels (10).

While previous research focused on the use of simple cross-tabulation (9), the Gini coefficient (11), and generalized linear models (12); they were often not sufficient to disentangle the complexity of the movements of doctors. To understand the spatial scales and explanatory factors associated to doctors' movements better, in addition to the traditional statistical analyses listed above, we have also employed a

For peer review only - http://bmjopen.bmj.com/site/about/guidelines.xhtml

new method, the Leslie matrix (usually applied in biology). We will show that our methods provide identification of the modal movement distances (short to long), their differences over time and the demographic and socio-economic factors associated with these scales.

Data and Methods

The data was provided from the UK Medical Education Database (UKMED) (project number UKMEDP44) and included 97,932 individuals who started medical school between 2002 and 2015. Of these, 871 had completed medical school and foundation training, and started or completed specialty training by the end of the study period (Supplementary File: Supplementary Information 1 and 2). We use the terms 'students' or 'medical students' to refer to those attending a medical school, and 'junior doctors' for those in foundation or specialty training. Since the dataset is only available up to 2015, the individuals for whom we have information for all three training stages are those who commenced medical school between 2002 and 2009. For all 97,932 students, the distances from the family home to medical school are available. Only individuals whose nationality was English, Welsh, Scottish or Northern Irish and whose medical degree was awarded from a UK university were considered in this study. The distances between the family home and the medical school considered only the medical school student's first choice, and therefore, subsequent changes are excluded. Out of 97,932 students, 5,093 (5.2%) changed medical schools. The 871 individuals include only those for whom the full training pathway was available without changes. 45 additional junior doctors who, at some point, were not in training or changed location during the foundation and specialty training were excluded. The analyses included 36 medical schools and all the hospitals providing training to the doctors across the UK.

This data was the full standardised data available at UKMED at that time. For each individual, the distances between each career stage (family home to medical school, medical school to foundation, foundation to specialisation) and other variables were provided (Table 1). For each indivdual, the distances were not summed (i.e., from the family home up to specialisation) and neither calculated between non-subsequent career stages (i.e., from the family home to foundation or specialisation or from medical school to specialisation). We used the term 'ethnicity' as per UKMED database, although there are on-going debates about its appropriate use (13) in particular for medical studies (14).

Six methods were used to investigate the movements of medical students across the UK. Four of these methods were integrated to investigate the movement distances and their patterns across the three stages and years: the Leslie model (known as a Leslie matrix), principal component analysis (PCA), Gini coefficient and chi-square test. Generalized linear models (GLMs) and variable selection methods were employed to evaluate the factors influencing the movements from family home to medical school (which

For peer review only - http://bmjopen.bmj.com/site/about/guidelines.xhtml

provide the largest data) at both individual and geographically aggregated levels. In practice, the first four analyses (Leslie, Gini, PCA and chi-square test) were applied to the 871 records containing the full pathways; while the Poisson GLMs and variable selection methods were applied on the whole 97,932 movements from family home to medical school.

The Leslie matrix is mostly applied to biological and ecological problems (15). The method is used to model population changes over time, including migration (16). It is a symmetric matrix with positive entries, where matrix cells contain the number of individuals passing from one stage to another. In this context, the stages are the education stages (medical school, foundation, specialisation). The matrix has been nested with distances, therefore for each stage, the number of students moving from one stage to the next is disaggregated by distance categories (17) (full method described in the Supplementary File: Supplementary Information 3). Since time is not integrated with space, the Leslie matrix is a simplified stage-only version. The lag distance applied is 25 km (0-25 km, 25-50 km, etc…). This lag allows for the identification of patterns at finer scales not possible if using a larger lag. Only the 871 individuals who completed all three training stages, were considered in the Leslie matrix construction.

The simplified stage-only Leslie matrix was analysed by employing a principal component analysis to evaluate the most important movements' distance categories among all training stages. This approach has been proposed by Usher and Williamson (17) and the resulting eigenvectors can be compared through chi-square tests to evaluate similarities and dissimilarities in the most important movements over years (18). In other words, annual Leslie matrices were created, and the relative first principal components (containing the largest amount of explained variance) for each year were extracted and compared in pairs by chi-square test.

To confirm equality or inequality in medical students and junior doctors (871 individuals) movement distance categories, the Gini coefficient has been calculated which has been used in similar researches (11). The Gini coefficient varies between 0 and 1 where 0 implies complete equality, and 1 complete inequality. The coefficient is derived from the Lorenz curve. When the Gini coefficient is 0, the Lorenz curve follows the line of equality, which is a straight diagonal line. If the distribution exhibits any inequality, the Lorenz curve falls below the line of equality. The Gini coefficient is the ratio between the Lorenz curve and the equality line. The Lorenz curve is the line passing through the points of cumulative percentage of the population (on the x-axis) and cumulative percentage of distances (on the y-axis).

Finally, a Poisson generalised linear model was applied individually on the distance (outcome) covered by each individual student from family home to medical school (therefore on the full 97,932 students). Distance was assumed a count data with discrete and non-negative values. The model explanatory factors were gender, ethnicity, and various variables representing the socio-economic status of the students and their parents (full list presented in Table 1). In addition, in the second Poisson generalised linear model,

For peer review only

For peer review only - http://bmjopen.bmj.com/site/about/guidelines.xhtml

the number of students moving from the county of family home to the county of medical school was used as outcome. Since the data is aggregated by county, the model included the distance between training and family home counties and the modal values of each explanatory variable for both the departing (family home) and arriving (training) county.

Before running the individual and county-level multivariable Poisson generalised linear models, selection of the explanatory variables was carried out by univariate modelling and stepwise selection (19) since all the explanatory variables are categorical. The variable selection approach included the following steps:

1) fit univariate models with each potential exploratory variable (Table 1);
2) on each univariate model a log-likelihood ratio test (comparing with the null model) is performed using the function poisson.anova in R-cran software(20) ;
3) the explanatory variable of the univariate model was included in the multivariable model if it caused the univariate model to be significantly different (at a significant level of 0.05) from the null model;
4) the multivariable model was then inputted in a stepwise selection method (function stepAIC in R-cran software(21)), with both forward and backward selection; the returned model with the lowest Akaike Information Criteria (AIC) (22, 23) was selected.

AIC was also employed to compare the Poisson regression model with a negative binomial model, which can account for overdispersion. The former yielded a lower AIC (indicating a better fit) than the negative binomial model, and for this reason, only the Poisson regression results are shown. The study was performed using Strengthening the Reporting of Observational studies in Epidemiology (STROBE) guidance(24).

Patient and Public involvement

Patients or the public were not involved in the design, or conduct, or reporting, or dissemination plans of our research.

For peer review only - http://bmjopen.bmj.com/site/about/guidelines.xhtml

188 Table 1: List of variables used in the analyses and their definitions. * Individual Student-related variables;
189 ** Individual student's parents-related variables. Source: UKMED data dictionary(25).

Variable	Description	Categories
Bursary*	Whether the individual is in the UKCAT Bursary table.	Yes<br>No
Distance*	Coded as the travel distance between centroids.	0 = from the county of family home to the county of the medical school; 1 = from home postcode to the medical school postcode; 2 = from medical school postcode to foundation training postcode; 3 = from foundation training postcode to specialty training postcode.
Domicile County*	The county the student family home is.	
Ethnicity*	Level 1 and Level 2 classification of ethnic groups as in 2011 Census.	Full list in Supplementary File: Supplementary Information 4.
BME*	Black and Minority Ethnic (BME).	0 = White<br>1 = BME
Gender*	The gender of the student.	0 = Male<br>1 = Female
Medical School County*	The county the medical school is in.	
Qualification*	The highest qualification the student has on entry to medical school.	1 = no formal qualification/not known;<br>2 = level 2 and below;<br>3 = level 3 including A levels and higher.
School Type*	Type of school the student attended between the ages of 11 and 16.	1 = independent/Private Fee Paying School; 2 = grammar School (selective); 3 = academy/Secondary School; 4 = further Education College / Sixth Form College; 5 = other.
Socio-Economic Classification*	Socio-economic background of students aged 21 and over at the start of their course, or for students under 21 the socio-economic background of their parent, step-parent or guardian who earns the most is returned. It is based on occupation, and if the parent or guardian is retired or unemployed, this is based on their most recent occupation.	1 = higher managerial & professional occupations; 2 = lower managerial & professional occupations; 3 = intermediate occupation; 4 = small employers & own account workers; 5 = lower supervisory & technical occupations; 6 = semi-routine occupations; 7 = routine occupations;8 = never worked & long-term unemployed; 9 = not classified.
Deprivation**	The IMD is the official measure of relative deprivation in England. It is based on 39 separate indicators, organised across seven domains of deprivation (income; employment, health deprivation and	1 = most deprived; 2; 3; 4; 5 = least deprived.

For peer review only - http://bmjopen.bmj.com/site/about/guidelines.xhtml

	disability; education, skills training; crime; barriers to housing and services; and living environment. The full rank is divided in five quintiles, with quintile 1 containing the most deprived or poor and quintile 5 containing the least deprived or most affluent.	
Income Support**	Whether the student's home has had any income support during their time at school.	Yes<br>No
Occupation**	Occupation of the household reference person. This is the person responsible for owning or renting or who is otherwise responsible for the accommodation. In the case of joint householders, the person with the highest income takes precedence. Where incomes are equal, the oldest person is taken as household reference. Based on the National Statistics Socio-Economic.	1 = managerial and professional occupations; 2 = intermediate occupations; 3 = small employers and account workers; 4 = lower supervisory and technical occupations; 5 = semi-routine and routine occupations.
Occupation individual**	Socioeconomic class occupation description of parent 1 and parent 2 from The UK Clinical Aptitude Test (UKCAT) questionnaire.	1 = modern professional (e.g. teacher, nurse, social worker, artist, police officer (sergeant or above)); 2 = clerical & intermediate (e.g. secretary, call centre agent, nursing auxiliary, nursery nurse); 3 = senior managers or administrators (e.g. finance manager, chief executive); 4 = technical & craft (e.g. motor mechanic, plumber, printer, tool maker, gardener, train driver, fitter); 5 = semi-routine manual & service (e.g. postal / farm worker, security guard, catering/sales assistant); 6 = routine manual & service (e.g. HGV driver, cleaner, porter, sewing machinist, bar staff, labourer); 7 = middle or junior managers (e.g. office / retail / bank / restaurant / warehouse manager, publican); 8 = traditional professional (e.g. accountant, solicitor, medical practitioner, scientist, civil servant); 9 = never worked; 10 = don't know or prefer not to say.

Results

The mean and standard deviation of the distances between each training stage are shown in Table 2. The

For peer review only - http://bmjopen.bmj.com/site/about/guidelines.xhtml

shorter, although more dispersed, distances are from the medical school to foundation programme training.

The principal component analysis of the simplified stage-only Leslie matrix, applied to only the individuals for which the full pathway is known (871 individuals), found that out of 40 principal components (one for each distance category), the first principal component (distances up to 25 km) explained 41% of the variance in the movements' distances, the second 13% (25-50 km) and the third 8% (50-75 km) (Supplementary File: Supplementary Information 5). The highest proportion of individuals (54%) concentrates in the shorter movement distances (up to 50 km) than longer movement distances. These results have been confirmed by the value of the Gini Coefficient for the Leslie matrix, which was 0.936 (Supplementary File: Supplementary Information 6) meaning inequality on the distribution of movements among all categories and stages, with modal distances at a short scale. These results indicate that most of the individuals stayed close to their home or training organisation.

Table 2. Summary statistics for the three career stage movements in the full dataset.

Distances	Mean (km)	Standard deviation (km)
From family home to medical school	173.8	155.6
From medical school to foundation training location	126.8	226.9
From foundation to specialty training location	161.8	216.8

The proportions of movements among the distance categories are heterogeneous in time. These differences have been tested by the chi-square test applied to paired first principal components of annual Leslie matrices (from 2002 to 2009 – where 2009 is the last year for which we have a full pathway for students). Figure 1 shows that 2002 proportion of movements are significantly different from most of the rest of the years (in red) at a significant level of 0.05, apart from 2007. 2004 was similar only to 2003 and 2009; 2006 to 2005, 2007 and 2009; and 2008 to 2005, 2007 and 2009. The year that was similar to most of all the other years is 2009, which was different only to 2002. Notably, the years with longest distances where 2007 and 2008 where the majority of individuals travelled distances up to 100-125 km (Supplementary File: Supplementary Information 5).

To evaluate determinants of the movement from family home to medical school at both individual and

For peer review only - http://bmjopen.bmj.com/site/about/guidelines.xhtml

aggregated (by county) level, generalised linear models were employed within a variable selection framework, the latter aimed at selecting the model with the lowest AIC or in other words the best model fitting.

Table 3: Poisson generalised linear model results for individual movements. Occ: occupation of the household reference; REF: reference category.

Variable	Coefficient	Standard Error	*p-value*
Intercept	5.197	0.015	< 0.001
Occ. managerial	REF		
Occ. intermediate	-0.086	0.002	< 0.001
Occ. small employers	-0.078	0.002	< 0.001
Occ. lower supervisory	-0.111	0.003	< 0.001
Occ. semi-routine	-0.121	0.003	< 0.001
Deprivation (Level 1)	REF		
Deprivation (Level 2)	0.011	0.001	< 0.001
Deprivation (Level 3)	-0.036	0.001	< 0.001
Deprivation (Level 4)	-0.069	0.001	< 0.001
Deprivation (Level 5)	-0.211	0.002	< 0.001
African	0.036	0.015	0.016
Arab	-0.244	0.015	< 0.001
Bangladeshi	-0.262	0.015	< 0.001
British	0.101	0.015	< 0.001
Caribbean	-0.016	0.016	0.325
Chinese	0.063	0.015	< 0.001
Gypsy or Irish Traveler	0.180	0.042	< 0.001
Indian	-0.157	0.015	< 0.001
Irish	0.347	0.015	< 0.001
Pakistani	-0.222	0.015	< 0.001
White and Asian	0.075	0.015	< 0.001
White and Black African	0.134	0.016	< 0.001
White and Black Caribbean	0.092	0.016	< 0.001
Other Asian background	-0.197	0.015	< 0.001

For peer review only

For peer review only - http://bmjopen.bmj.com/site/about/guidelines.xhtml

Other Black background	0.035	0.021	0.089
Other Mixed background	-0.046	0.015	0.002
Other White background	0.108	0.015	< 0.001
Other Ethnic group	-0.170	0.015	< 0.001
Not stated	0.081	0.022	< 0.001

At individual level, the best fitting model of the distances travelled by students was obtained with ethnicity sub-group (Level 2 information), student's parents' occupation and deprivation (Table 3). All the classes of deprivation and parent occupation were found to be statistically significant. In terms of deprivation, compared to the most deprived, the least deprived levels (3 to 5) travelled a shorter distance when they moved from their family home to medical school. Compared to students with parents in managerial and professional occupation, the students with other occupations travelled shorter distances (Table 3). In terms of the ethnicity variable, it was possible to identify that Arab, Bangladeshi, Indian, Pakistani, 'other Asian background', 'other mixed background' and 'other ethnic group' students travelled shorter distances to reach the medical school than British, Chinese, Gypsy or Irish traveller, Irish, Scottish, White and Asian, White and Black Caribbean, White and black African, and 'other white background' students. Within these two groups of ethnicities distinguished for their relationships with travelled distance, a three-fold difference was found between British and African in their longer movements (with British moving further), while a 1.6-fold difference was found between Arab and Indian in their shorter movements (with Arab staying closer).

Table 4: Poisson generalized liner model results for county-aggregated data. Departing = county of family home; arriving = county with the medical school.

Variable	Estimate	Standard Error	*p-value*
Intercept	-0.963	0.068	< 0.001
Distance between counties	-0.006	0.000	< 0.001
BME departing county (No)	REF		
BME departing county (Yes)	-1.204	0.042	< 0.001
Gender departing county (Male)	REF		
Gender departing county (Female)	2.691	0.029	< 0.001
Qualification departing county (below level 3/not known)	REF		
Qualification departing county (level 3)	0.085	0.004	< 0.001
Deprivation departing county (level 1)	REF		

For peer review only

Deprivation departing county (above level 1)	-0.454	0.005	< 0.001
Deprivation arriving county (level 1)	REF		
Deprivation arriving county (above level 1)	-0.483	0.006	< 0.001
Students socio-economic (higher managerial)	REF		
Students socio-economic (lower and below)	-0.183	0.021	< 0.001

The best fitting model by county (Table 4) confirms the importance of deprivation in the number of students moving from family home to medical school counties. Distance between counties was an important factor for the number of students moving from one county to the other, with medical schools having more students coming from neighbouring counties than for counties further away (negative association with distance). The rest of the most significant variables were all associated with the departing county: BME, female students, highest qualification at entry, and SEC (Table 4). In particular, counties with higher proportions of BME or with higher deprivation or socio-economic status were associated with fewer individuals moving. While counties with a larger proportion of female students or higher qualification at entry were associated with larger number of movements.

For peer review only

Discussion

This research found that the largest proportion of individuals travelled within 25 km to the next stage of professional development, with most of the movements being statistically significant at less than 50 km, although not for every year under study. In fact, in the years of the financial crisis (2007-2008) (26) individuals within the three training stages travelled further (up to 125 km). Although this study was not aimed at investigating the effect of the financial crisis on UK medical students' movements, it is certainly the first study proposing this hypothesis. A study on medical students in Spain reported that students focused more on career prospects than lifestyle in response of the economic crisis (27), when choosing their specialty. If this was the reason for longer movements by UK medical students and junior doctors it is something that will need to be ascertained.

The up-to 50 km modal distance travelled by UK-born medical students and junior doctors for all the years between 2002 and 2009 is reported here for the first time. A 2019 GMC report that focused on doctors who gained their UK Primary Medical Qualification between 2012 and 2018 found that almost a quarter (23%) of doctors in 2019 were working within 10 miles (16 km) of where they studied at medical school (28). However, a third of graduates moved more than a hundred miles.

Despite the absence of distance information, a previous study (on a restricted number of doctors and obtained by questionnaire) reported that, between 1974 and 2008, 36% of the doctors stayed in the same

For peer review only - http://bmjopen.bmj.com/site/about/guidelines.xhtml

region as their family home during medical school and 48% undertook specialist training in the same region as their medical school (29). A more recent observational study that focused on doctors graduating between 2012 and 2014, found that the majority of doctors trained in foundation schools that were close to their family homes (30).

We found strong inequality in the movements – e.g. the presence of dominant movement distance categories compared to others. A previous study using Gini coefficient applied to movements for general practitioners between 1974 and 2003 (7) found greater equality (Gini coefficient between 0.4 and 0.5) than the present research. A direct comparison between the two studies cannot be done, but the increase in medical student number supply since 1974 (7) could have caused a more pronounced movements inequality (as we found) than previously.

The main factors associated to students' movement distances and student counts from family home to medical school are very similar at both individual and aggregated – by county – levels. The most important factors were socioeconomic status, deprivation score and ethnicity (BME, Ethnicity group and sub-groups). In our analyses, increased deprivation was found to be associated with both fewer doctors moving and shorter distances moved, while more students with higher degrees at entry to medical school or with parents with professional occupation tended to move further away. In previous literature, Primary Care Trusts (PCT) in England are seen to be attractive if there is less crime in the area, or better schools and opportunities (10). Similarly those students with parents in NSSEC1 (professional occupations) were less likely to work in deprived areas compared to those in NSSEC5 (routine occupations) (31). Regarding the ethnicity, one study reported that doctors from non-white ethnic backgrounds were more likely to choose to work in a foundation trust closer to their family home (29). We found the same results though not all the non-white ethnic groups are staying closer. In fact, we were able to distinguish the group moving closer to their family home, mainly from middle east and Indian background from those that moved further (the rest of the groups), although our analyses do not account for the potential geographical clustering of ethnic minorities in the UK (32) which can potentially bias the results. Personal factors such as ethnicity, education and gender were all significant contributing factors to the movement of doctors at both individual and county levels (33).

Consideration of the structural elements of our societies, such as deprivation and demography (e.g. ethnicity), will require different type, spatial scale and economic effort for improving the distribution of doctors in the UK. The NHS proposed a 'rural weighting' payment in England which would aim to attract doctors and GPs to rural areas with a financial incentive (34, 35). This may be equally applicable in areas of high deprivation. Payments of this type could also be increased in line with the length of service thereby increasing retention of medical practitioners within rural or deprived areas. Another potential solution is expanding widening access to medical schools and create new medical schools in areas

For peer review only - http://bmjopen.bmj.com/site/about/guidelines.xhtml

underserved given the tendency of the students to stay local to their training institution (36). In Scotland it has been found that there is a link between childhood background, as defined by parental ocioeconomic status and being in more remote residence, and the population the GPs are likely to serve(31). The authors suggested that GPs residing in more rural areas and those who experienced greater deprivation during their childhood tended to work in settings that reflected these characteristics. While this should not be presumed from these students, World Health Organisation recommendations emphasise the need to create more opportunities, such as locating educational facilities closer to rural areas, and providing incentives, such as benefits or direct payments, to medical students and doctors to serve in rural areas (37). However, recent literature indicates that outcomes of financial incentives are limited or unclear (38). Family-unit and community considerations are often the predominant factors influencing doctors' movements (39).

The main limitation of our study is the absence of historical mapping for all distances between medical schools, foundation, and specialty training facilities. Currently, this information is unavailable, but it is crucial to test alternative hypotheses regarding the likelihood of movement distance in relation to the availability of training facilities in the surrounding area, weighted by the number of positions available at the training location.

Other limitations are also present. This study includes information up to 2015, which may not reflect recent changes in medical education or workforce dynamics. Students who left their medical education or moved between training locations are not included in the dataset; therefore, the results are not generalisable for this group of students. We use the term 'ethnicity' as per the UKMED database, based on the categorisation adopted by the Office for National Statistics. However, this may not be appropriate due to limitations in the way this variable is defined and measured.

Finally, the present study did not consider international medical graduates working in the UK, UK-born doctors working abroad, or records pre-2002. Data for these doctors were not available. A new study (NIHR134540) (40) is investigating why doctors work where they work and how this is influenced by the organisation of the healthcare system, the social and physical environment where the future doctors grow up and move as well as their individual beliefs and choices. This new study which uses mixed methods and a larger dataset than any previously should increase the understanding around doctors movements from the doctor's perspectives as well and not only from data records. We hope that our study and the ones currently active will identify the best interventions to improve the underdoctoring and maldistribution of doctors across the UK.

Author Contributions:

CM and LS conceived and designed the study. LH, DS, JR CM and LS discussed and drafted the study protocol. LH and BF screened and selected the articles. LH and BF analysed the data. LH,

For peer review only - http://bmjopen.bmj.com/site/about/guidelines.xhtml

For peer review only

BF and LS interpreted the results. LH, BF and LS drafted the manuscript. All authors critically revised the manuscript, contributed to revising the manuscript and had final responsibility for the decision to submit for publication. DS, JR, CM and LS supervised the study. The corresponding author attests that all listed authors meet authorship criteria and that no others meeting the criteria have been omitted.

Competing interests:

CM is medical director and director of education and standards at the General Medical Council. Views expressed in this article do not necessarily reflect those of the General Medical Council.

Data sharing

The data for this research was accessed through the cloud from the UK Medical Education Database (UKMED), specifically from the UKMEDP044 extract generated on 01/06/2018. The availability of the data used in this study is through an application process to UKMED, and more information can be found on their website: https://www.ukmed.ac.uk/. Approved for publication on 30/06/2023.

Funding

LS is supported by the NIHR Health and Social Care Delivery Research (HSDR) programme (NIHR134540) and North West Cancer Research UK (LI2021SEDDA). The views expressed are those of the author(s) and not necessarily those of the NIHR or the Department of Health and Social Care.

Ethical approval

The Medical Schools Council agreed that all approved applications for research projects using data exclusively held by UKMED would meet the criteria for a blanket exemption from the need to apply for ethics approval that would be recognised by ethics committees relevant to the UK medical schools. A letter was written from QMUL Ethics of Research Committee on behalf of all UK medical schools to confirm ethics exemption (available at chrome-extension://efaidnbmnnnibpcajpcglclefindmkaj/https://www.ukmed.ac.uk/documents/UKMED_research_projects_ethics_exemption.pdf).

Acknowledgments

We are grateful to UKMED for the use of these data. However, UKMED has no responsibility for the analysis or interpretation of their data. The data includes information derived from that collected by the

For peer review only

For peer review only - http://bmjopen.bmj.com/site/about/guidelines.xhtml

Higher Education Statistics Agency Limited ("HESA") and provided to the GMC ("HESA Data"). Source: HESA Student Records 2002/2015 Copyright Higher Education Statistics Agency Limited. The Higher Education Statistics Agency Limited makes no warranty as to the accuracy of the HESA Data, cannot accept responsibility for any inferences or conclusions derived by third parties from data or other information supplied by it.

References

1. Rolewicz L, Palmer B, Lobont C. The NHS workforce in numbers: Nuffield Trust; 2022 [Available from: https://www.nuffieldtrust.org.uk/resource/the-nhs-workforce-in-numbers.
2. Government S. Health Workforce [Available from: https://www.gov.scot/policies/health-workforce/.
3. Government W. Staff directly employed by the NHS: as at 30 September 2022 2022 [Available from: https://www.gov.wales/staff-directly-employed-nhs-30-september-2022-html.
4. Health Do. Northern Ireland health and social care (HSC) workforce statistics December 2022 2023 [Available from: https://www.health-ni.gov.uk/publications/northern-ireland-health-and-social-care-hsc-workforce-statistics-december-2022.
5. McCarthy N. The World's Biggest Employers Jersey City, NJ, US: Forbes; 2015 [Available from: https://www.forbes.com/sites/niallmccarthy/2015/06/23/the-worlds-biggest-employers-infographic.
6. Brice J, Corrigan O. The changing landscape of medical education in the uk. . Medical Teacher. 2010;32(9):727-32.
7. Hann M, Gravelle H. The maldistribution of general practitioners in england and wales: 1974-2003. The British journal of general practice : the journal of the Royal College of General Practitioners. 2004;54(509).
8. British Medical Association. NHS medical staffing data analysis2023 06/07/2023. Available from: https://www.bma.org.uk/advice-and-support/nhs-delivery-and-workforce/workforce/nhs-medical-staffing-data-analysis.
9. Parkhouse J, Lambert T. Home, training and work: Mobility of british doctors. 1997;31:399-407.
10. Goddard M, Gravelle H, Hole A, Marini G. Where did all the gps go? increasing supply and geographical equity in england and scotland. Journal of health services research & policy,. 2010;15(1):28-35.
11. Morita T, Tanimoto T, Morita M, Tsubokura M, M. K. Trend in unequal geo-graphical distribution of doctors by age and sex in japan from 2004 to 2014. Public Health. 2018;159:95-8.
12. Ide H, Doi S, Atarashi H, Fujita S, Koike S. The distance and chance of lifetime geographical movement of physicians in japan: an analysis using the age-period-cohort model. Human Resources for Health. 2018;16(1).
13. Flanagin A, Frey T, Christiansen SL. Updated Guidance on the Reporting of Race and Ethnicity in Medical and Science Journals. Jama-J Am Med Assoc.

For peer review only - http://bmjopen.bmj.com/site/about/guidelines.xhtml

2021;326(7):621-7.
14. Martinez RAM, Andrabi N, Goodwin AN, Wilbur RE, Smith NR, Zivich PN. Conceptualization, Operationalization, and Utilization of Race and Ethnicity in Major Epidemiology Journals, 1995-2018: A Systematic Review. Am J Epidemiol. 2023;192(3):483-96.
15. Monte L. Characterisation of a nonlinear leslie matrix model for predicting the dy- namics of biological populations in polluted environments: Applications to radioecology. Ecological Modelling. 2013;248:174-83.
16. Leslie P. On the use of matrices in certain population mathematics. 1945;33:183-212.
17. Usher M, Williamson M. A deterministic matrix model for handling the birth, death, and migration processes of spatially distributed populations. . 1970;26:1-12.
18. Freund R. Symmetric matrices and eigendecomposition2014. Available from: http://s3.amazonaws.com/mitsloan-php/wp-faculty/sites/30/2016/12/15032137/Symmetric-Matrices-and-Eigendecomposition.pdf.
19. Upton G, Cook I. Forward selection 2014 [Available from: http://www.oxfordreference.com/view/10.1093/acref/9780199679188.001.0001/acref-9780199679188-e-624.
20. Tsagris M, Papadakis M. Taking R to its limits: 70+ tips. PeerJ Preprints. 2018;6.
21. Ripley BD. Modern applied statistics with S: springer; 2002.
22. Symonds M, Moussalli A. A brief guide to model selection, multimodel inference and model averaging in behavioural ecology using akaike's information criterion. Behavioral Ecology and Sociobiology. 2011;65(1):13-21.
23. Gelman A. Discussion paper: Analysis of variance - why it is more important than ever. The Annals of Statistics. 2005;33(1):1-33.
24. Vandenbroucke JP, von Elm E, Altman DG, Gotzsche PC, Mulrow CD, Pocock SJ, et al. Strengthening the Reporting of Observational Studies in Epidemiology (STROBE): explanation and elaboration. Epidemiology. 2007;18(6):805-35.
25. UKMED - 2018 V1. London: UKMED; 2018. Data Dictionary.
26. Lindstrom M, Giordano GN. The 2008 financial crisis: Changes in social capital and its association with psychological wellbeing in the United Kingdom - A panel study. Soc Sci Med. 2016;153:71-80.
27. Harris JE, Lopez-Valcarcel BG, Ortun V, Barber P. Specialty choice in times of economic crisis: a cross-sectional survey of Spanish medical students. Bmj Open. 2013;3(2).
28. GMC. The state of medical education and practice in the UK 2019 [Available from: https://www.gmc-uk.org/-/media/documents/the-state-of-medical-education-and-practice-in-the-uk---workforce-report_pdf-80449007.pdf.
29. Goldacre M, Davidson J, Maisonneuve J, Lambert T. Geographical movement of doctors from education to training and eventual career post: UK cohort studies. J R Soc Med. 2013;106(3):96-104.
30. Kumwenda B, Cleland JA, Prescott GJ, Walker KA, Johnston PW. Geographical mobility of UK trainee doctors, from family home to first job: a national cohort study. BMC Med Educ. 2018;18(1):314.
31. Dowell J, Norbury M, Steven K, Guthrie B. Widening access to medicine may improve general practitioner recruitment in deprived and rural communities: survey of gp origins and current place of work. BMC Medical Education. 2015;15(1).

For peer review only - http://bmjopen.bmj.com/site/about/guidelines.xhtml

32. Shaw B, Menzies L, Bernardes E, Baars S, Nye P, Allen R. Ethnicity, gender and social mobility. London: Social Mobility Commission. 2016.
33. Dussault G, Franceschini M. Not enough there, too many here: understanding geographical imbalances in the distribution of the health workforce. Human Resources for Health. 2006.
34. RSN RSN. Shortage of rural doctors puts patients' health at risk. 2017 [Available from: http://rsnonline.org.uk/shortage-of-rural-doctors-puts-patients-health-at-risk.
35. Rolewicz L, Palmer B. Placed at a premium? The use of recruitment and retention pay supplements to address staffing shortfalls: Nuffield Trust; 2022 [Available from: https://www.nuffieldtrust.org.uk/research/placed-at-a-premium.
36. Verma P, Ford JA, Stuart A, Howe A, Everington S, Steel N. A systematic review of strategies to recruit and retain primary care doctors. BMC Health Serv Res. 2016;16:126.
37. WHO. WHO guideline on health workforce development, attraction, recruitment and retention in rural and remote areas. World Health Organization Geneva; 2021.
38. Swami M, Scott A. Impact of rural workforce incentives on access to GP services in underserved areas: Evidence from a natural experiment. Soc Sci Med. 2021;281:114045.
39. Holloway P, Bain-Donohue S, Moore M. Why do doctors work in rural areas in high-income countries? A qualitative systematic review of recruitment and retention. Aust J Rural Health. 2020;28(6):543-54.
40. NIHR. HSDR Funding Committee Public Minutes March 2022 2022 [Available from: https://www.nihr.ac.uk/documents/hsdr-funding-committee-public-minutes-march-2022/30467.

For peer review only

For peer review only - http://bmjopen.bmj.com/site/about/guidelines.xhtml

494 Figure Legend

495 Figure 1: Chi-square p-values from comparison of annual first components of the
496 Leslie matrix. p-values below 0.05 are shown in red.

For peer review only

For peer review only - http://bmjopen.bmj.com/site/about/guidelines.xhtml

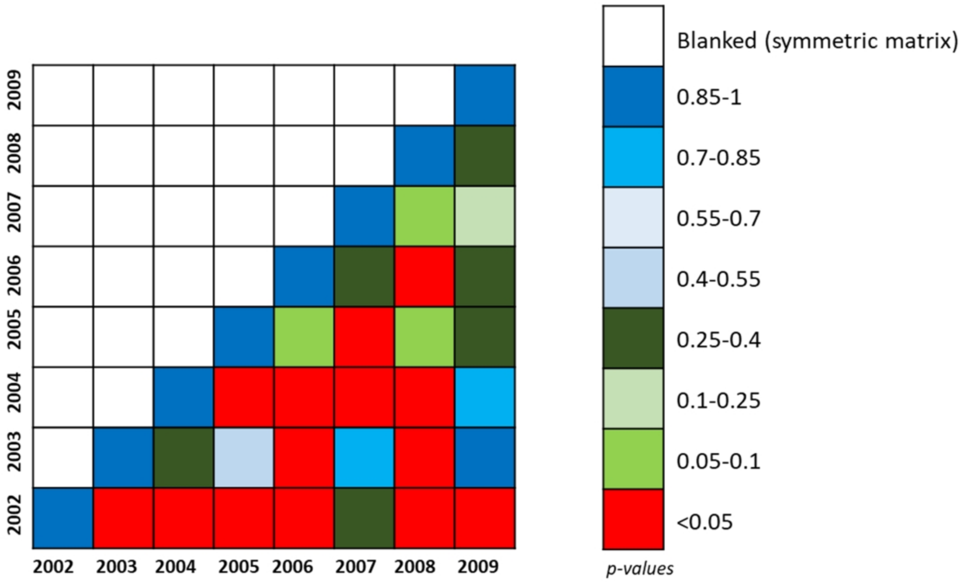

Figure 1. Chi-square p-values from comparison of annual first components of the Leslie matrix. p-values below 0.05 are shown in red.

1200x800mm (96 x 96 DPI)

Determining the distance patterns in the movements of future doctors in UK between 2002 and 2015: a retrospective cohort study

Lucy A. Hitchings, Benjamin G. Fleet, Daniel Smith, Jonathan Read, Colin Melville, Luigi Sedda

Supplementary File

Contents

Supplementary information 1: Table S1. Cohort of medical students and junior doctors considered int the study. Foundation year one (F1) and Specialty year one (S1) numbers are referred to individuals that started the medical school between 2002 and 2015. 2

Supplementary information 2: Table S2. Number of junior doctors starting S1 between 2011 and 2015 (second row) and contribution by medical school year. 3

Supplementary information 3: A simplified stage only Leslie Matrix 4

Supplementary information 4: UKMED Ethnicity classification 5

Supplementary information 5: Principal component analysis results 6

Supplementary information 6: Lorenz curve 8

For peer review only

Supplementary information 1: Table S1. Cohort of medical students and junior doctors considered int the study. Foundation year one (F1) and Specialty year one (S1) numbers are referred to individuals that started the medical school between 2002 and 2015.

Year	Number medical students starting medical school	Number of junior doctors in F1	Number of junior doctors in S1
2002	6640	0	0
2003	6824	0	0
2004	7190	0	0
2005	7201	0	0
2006	7145	0	0
2007	7042	0	0
2008	7193	0	0
2009	7100	4487	0
2010	7130	5331	0
2011	7193	5684	137
2012	6936	6494	309
2013	6751	6378	236
2014	6810	6752	114
2015	6777	6597	75
Totals	*97932*	*41723*	*871*

For peer review only

Supplementary information 2: Table S2. Number of junior doctors starting S1 between 2011 and 2015 (second row) and contribution by medical school year.

Year junior doctors started S1	2011	2012	2013	2014	2015
Number of junior doctors in S1	137	309	236	114	75
Proportion of junior doctors in S1 (second row) that started medical school in 2002	14.39	3.39	2.65	2.75	2.78
Proportion of junior doctors in S1 (second row) that started medical school in 2003	75.76	29.49	11.95	14.68	6.94
Proportion of junior doctors in S1 (second row) that started medical school in 2004	6.82	58.98	18.14	28.44	8.33
Proportion of junior doctors in S1 (second row) that started medical school in 2005	2.27	7.12	63.72	26.61	31.94
Proportion of junior doctors in S1 (second row) that started medical school in 2006	0.76	0.68	2.21	23.85	20.83
Proportion of junior doctors in S1 (second row) that started medical school in 2007	0.00	0.34	1.33	0.00	22.22
Proportion of junior doctors in S1 (second row) that started medical school in 2008	0.00	0.00	0.00	0.92	3.39
Proportion of junior doctors in S1 (second row) that started medical school in 2009	0.00	0.00	0.00	2.75	3.57

For peer review only

Supplementary information 3: A simplified stage only Leslie Matrix

A distance matrix is a square matrix that consists of the distances between elements, where the elements are the three stages required to complete the medical career. Those three stages are medical school, foundation training and specialty training. Movements are defined as the distance covered by the students from and to postcodes of two successive stages. Movement distances are lagged at 25 km interval.

The following example describes the construction of the Leslie matrix for the first three distances (D1=0-25km, D2=25-50km and D3=50-75km). First consider students travelling from family home to medical school, they are grouped by distance along the matrix diagonal (matrix A):

$$A = \begin{bmatrix} D_1 & & \\ & D_2 & \\ & & D_3 \end{bmatrix}$$

For each of the students who travelled a particular distance onto medical school, each of these will also have moved a certain distance onto their respective foundation training. Starting from the students in D1, they are split between students that travelled 0-25 km (F1), 25-50 km (F2) and 50-75 km (F3). In the same way for students in D2 and D3 (matrices B).

$$B(D_1) = \begin{bmatrix} F_{1,1_{D_1}} & F_{1,2_{D_1}} & F_{1,3_{D,1}} \\ F_{2,1_{D_1}} & & \\ F_{3,1_{D_1}} & & \end{bmatrix}, \quad B(D_2) = \begin{bmatrix} & F_{1,2_{D_2}} & \\ F_{2,1_{D_2}} & F_{2,2_{D_2}} & F_{2,3_{D_2}} \\ & F_{3,2_{D_2}} & \end{bmatrix},$$

$$B(D_3) = \begin{bmatrix} & & F_{1,3_{D_3}} \\ & & F_{2,3_{D_3}} \\ F_{3,1_{D_3}} & F_{3,2_{D_3}} & F_{3,3_{D_3}} \end{bmatrix}$$

Since the matrices are symmetric, $F_{1,2}$ and $F_{2,1}$ have the same values. The same matrices can be created for the F1 distance students going to specialty training (C matrices):

$$C(F_{1,1}) = \begin{bmatrix} S_{1,1_{F1,1}} & S_{1,2_{F1,1}} & S_{1,3_{F1,1}} \\ S_{2,1_{F1,1}} & & \\ S_{3,1_{F1,1}} & & \end{bmatrix}$$

Where $S_{1,1}$ is the number of students that travelled 0-25km in all the stages, i.e. from home to medical school (D1), from medical school to foundation (F1) and from foundation to specialty training (S1). Finally, the Leslie matrix is the sum of matrix A with matrices B and C. This matrix is a symmetric matrix with positive entries and has the same structure as a Leslie matrix, although a Leslie matrix is formulated based on divisions such as stage and age, assuming only population flows in adjacent cells of the matrix. By considering only non-overlapping stages (Murray and Sandercock, 2020), flow to non-contiguous cells is possible (Usher and Williamson, 1970).

References

Murray, Dennis L., and Brett K. Sandercock, eds. Population ecology in practice. John Wiley & Sons, 2020.

Usher, M. B., and M. H. Williamson. "A deterministic matrix model for handling the birth, death, and migration processes of spatially distributed populations." Biometrics (1970): 1-12.

For peer review only

Supplementary information 4: UKMED Ethnicity classification

Level 1 (bold) and Level 2 Ethnicity.
https://www.ethnicity-facts-figures.service.gov.uk/style-guide/ethnic-groups

Asian or Asian British

- Indian
- Pakistani
- Bangladeshi
- Chinese
- Any other Asian background

Black, African, Caribbean or Black British

- African
- Caribbean
- Any other Black, African or Caribbean background

Mixed or multiple ethnic groups

- White and Black Caribbean
- White and Black African
- White and Asian
- Any other Mixed or multiple ethnic background

White

- English, Welsh, Scottish, Northern Irish or British
- Irish
- Gypsy or Irish Traveller
- Any other White background

Other ethnic group

- Arab
- Any other ethnic group

For peer review only

Supplementary information 5: Principal component analysis results

Table S3: Proportion of variance explained by the distance categories for all years and by each year from principal component analysis applied to all years and yearly Leslie matrices respectively.

km	Overall	2002	2003	2004	2005	2006	2007	2008	2009
0-25	40.98%	42.70%	40.07%	43.12%	45.34%	31.28%	28.55%	28.19%	43.45%
25-50	13.19%	1.32%	12.17%	15.84%	15.87%	10.82%	5.58%	4.72%	14.91%
50-75	7.84%	5.27%	6.47%	9.07%	9.83%	2.62%	6.01%	0%	0%
75-100	4.75%	5.09%	4.98%	5.25%	2.94%	8.12%	7.48%	6.17%	0%
100-125	8.47%	11.15%	7.34%	7.73%	8.36%	6.91%	12.70%	21.80%	0%
125-150	6.00%	9.83%	7.06%	3.07%	3.78%	15.86%	14.69%	7.72%	10.68%
150-175	1.11%	1.50%	1.29%	0.45%	1.23%	4.31%	1.11%	<0.1%	0%
175-200	1.35%	2.39%	1.61%	1.24%	0.37%	0.76%	2.53%	1.54%	6.69%
200-225	0.74%	0%	0.72%	0.57%	0.28%	2.01%	0.49%	4.64%	0%
225-250	1.17%	5.95%	2.06%	1.62%	0.64%	1.62%	0%	1.46%	4.59%
250-275	0.51%	2.26%	0.77%	0.21%	0.23%	0.51%	1.11%	1.13%	1.81%
275-300	0.36%	0%	0.72%	<0.1%	0.59%	0%	0%	0%	0%
300-325	1.34%	0.17%	3.97%	0.22%	0.63%	0.14%	1.15%	0.23%	0%
325-350	0.42%	1.20%	0.46%	0.40%	0.22%	<0.1%	1.11%	0.15%	0%
350-375	1.50%	1.36%	2.26%	1.69%	0.61%	3.88%	0%	3.08%	0%
375-400	0.78%	0%	1.24%	1.15%	0.43%	0%	2.18%	<0.1%	0%
400-425	0.56%	0%	1.01%	0.39%	0.41%	2.19%	<0.1%	1.07%	0%
425-450	0.58%	0%	0.44%	0.58%	0.78%	2.16%	0%	<0.1%	0%
450-475	4.43%	4.60%	2.32%	5.07%	4.20%	2.42%	5.66%	1.93%	0%
475-500	0.36%	1.19%	0.51%	0.26%	0.30%	1.01%	0%	1.48%	0%
500-525	0.33%	1.65%	0.27%	<0.1%	0.20%	0%	0%	1.47%	0%
525-550	<0.1%	0%	<0.1%	<0.1%	<0.1%	0.57%	1.07%	<0.1%	0%
550-575	0.27%	0%	0%	0%	0.20%	0.41%	4.04%	0.68%	0%
575-600	0.22%	0%	0.43%	<0.1%	0.14%	0.99%	0%	0%	0%
600-625	0.14%	0%	0%	0.21%	0.28%	0%	0%	0%	0%
625-650	<0.1%	0%	0%	0.18%	<0.1%	0%	1.08%	0%	0%
650-675	0.16%	0%	0.21%	<0.1%	0.20%	<0.1%	1.07%	0%	0%
675-700	0.11%	<0.1%	0%	<0.1%	0.20%	0%	0%	0%	7.90%
700-725	0.26%	1.13%	<0.1%	<0.1%	0.26%	0%	0.99%	1.39%	0%
725-750	0.42%	0%	0.65%	0.23%	0.41%	0%	1.10%	6.30%	0%
750-775	0.18%	0%	<0.1%	<0.1%	0.58%	<0.1%	0%	<0.1%	0%

For peer review only

775-800	0.71%	1.20%	0.44%	0.97%	<0.1%	<0.1%	0.23%	2.46%	0%
800-825	0.13%	0%	0%	0.19%	0.26%	0%	<0.1%	0%	0%
825-850	0.17%	0%	0.24%	0.18%	<0.1%	0%	0%	0%	0%
850-875	0.18%	0%	0%	0%	0.20%	0%	<0.1%	0%	9.97%
875-900	<0.1%	0%	<0.1%	0%	0%	1.34%	0%	2.15%	0%
900-925	<0.1%	<0.1%	0%	0%	0%	0%	0%	0%	0%
925-950	0%	0%	0%	0%	0%	0%	0%	0%	0%
950-975	<0.1%	0%	0.20%	0%	0%	0%	0%	0%	0%

For peer review only

Supplementary information 6: Lorenz curve

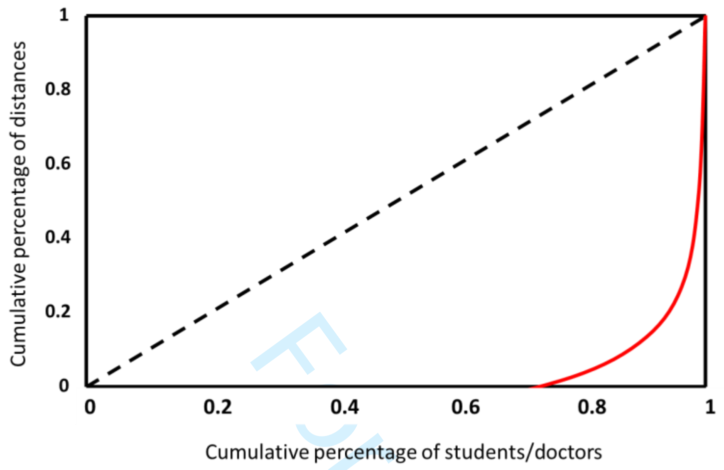

Figure S1: Lorenz curve of the Leslie Matrix. Equality (Gini coefficient equal to 0) is achieved when the Lorenz curve (bold black line) coincide with the diagonal. The diagonal represents the status where all distances have equal number of students. In this figure a strong inequality is shown, with the entire Lorenz curve laying on values above 0.7.

For peer review only

Reporting checklist for cohort study.

Based on the STROBE cohort guidelines.

Instructions to authors

Complete this checklist by entering the page numbers from your manuscript where readers will find each of the items listed below.

Your article may not currently address all the items on the checklist. Please modify your text to include the missing information. If you are certain that an item does not apply, please write "n/a" and provide a short explanation.

Upload your completed checklist as an extra file when you submit to a journal.

In your methods section, say that you used the STROBE cohortreporting guidelines, and cite them as:

von Elm E, Altman DG, Egger M, Pocock SJ, Gotzsche PC, Vandenbroucke JP. The Strengthening the Reporting of Observational Studies in Epidemiology (STROBE) Statement: guidelines for reporting observational studies.

		Reporting Item	Page Number
**Title and abstract**			
Title	#1a	Indicate the study's design with a commonly used term in the title or the abstract	1
Abstract	#1b	Provide in the abstract an informative and balanced summary of what was done and what was found	3
**Introduction**			
Background / rationale	#2	Explain the scientific background and rationale for the investigation being reported	4
Objectives	#3	State specific objectives, including any prespecified hypotheses	4
**Methods**			
Study design	#4	Present key elements of study design early in the paper	5
Setting	#5	Describe the setting, locations, and relevant dates, including periods of recruitment, exposure, follow-up, and data	5

For peer review only

		collection	
Eligibility criteria	#6a	Give the eligibility criteria, and the sources and methods of selection of participants. Describe methods of follow-up.	n/a no selection
Eligibility criteria	#6b	For matched studies, give matching criteria and number of exposed and unexposed	n/a no matched study
Variables	#7	Clearly define all outcomes, exposures, predictors, potential confounders, and effect modifiers. Give diagnostic criteria, if applicable	6 and 7
Data sources / measurement	#8	For each variable of interest give sources of data and details of methods of assessment (measurement). Describe comparability of assessment methods if there is more than one group. Give information separately for for exposed and unexposed groups if applicable.	5
Bias	#9	Describe any efforts to address potential sources of bias	2
Study size	#10	Explain how the study size was arrived at	n/a all database considered
Quantitative variables	#11	Explain how quantitative variables were handled in the analyses. If applicable, describe which groupings were chosen, and why	5 and 6
Statistical methods	#12a	Describe all statistical methods, including those used to control for confounding	
5 and 6			
Statistical methods	#12b	Describe any methods used to examine subgroups and interactions	5 and 6
Statistical methods	#12c	Explain how missing data were addressed	n/a no missing data
Statistical methods	#12d	If applicable, explain how loss to follow-up was addressed	n/a
Statistical methods	#12e	Describe any sensitivity analyses	
n/a Different models were tested (supplementary)			

For peer review only

Results

Participants	#13a	Report numbers of individuals at each stage of study—eg numbers potentially eligible, examined for eligibility, confirmed eligible, included in the study, completing follow-up, and analysed. Give information separately for for exposed and unexposed groups if applicable.	n/a
Participants	#13b	Give reasons for non-participation at each stage	n/a
Participants	#13c	Consider use of a flow diagram	

n/a

Descriptive data	#14a	Give characteristics of study participants (eg demographic, clinical, social) and information on exposures and potential confounders. Give information separately for exposed and unexposed groups if applicable.	n/a
Descriptive data	#14b	Indicate number of participants with missing data for each variable of interest	

n/a

Descriptive data	#14c	Summarise follow-up time (eg, average and total amount)	

n/a

Outcome data	#15	Report numbers of outcome events or summary measures over time. Give information separately for exposed and unexposed groups if applicable.	

9

Main results	#16a	Give unadjusted estimates and, if applicable, confounder-adjusted estimates and their precision (eg, 95% confidence interval). Make clear which confounders were adjusted for and why they were included	n/a Supplementary file
Main results	#16b	Report category boundaries when continuous variables were categorized	n/a
Main results	#16c	If relevant, consider translating estimates of relative risk into absolute risk for a meaningful time period	

n/a

For peer review only

Other analyses	#17	Report other analyses done—eg analyses of subgroups and interactions, and sensitivity analyses	n/a Supplementary file
**Discussion**			
Key results	#18	Summarise key results with reference to study objectives	10 and 11
Limitations	#19	Discuss limitations of the study, taking into account sources of potential bias or imprecision. Discuss both direction and magnitude of any potential bias.	2
Interpretation	#20	Give a cautious overall interpretation considering objectives, limitations, multiplicity of analyses, results from similar studies, and other relevant evidence.	10 and 11
Generalisability	#21	Discuss the generalisability (external validity) of the study results	10 and 11
**Other Information**			
Funding	#22	Give the source of funding and the role of the funders for the present study and, if applicable, for the original study on which the present article is based	12

Notes:

- 6a: n/a no selection
- 6b: n/a no matched study
- 10: n/a all database considered
- 12c: n/a no missing data
- 12e: n/a Different models were tested (supplementary)
- 16a: n/a Supplementary file
- 17: n/a Supplementary file The STROBE checklist is distributed under the terms of the Creative Commons Attribution License CC-BY. This checklist was completed on 06. July 2023 using https://www.goodreports.org/, a tool made by the EQUATOR Network in collaboration with Penelope.ai

For peer review only

BMJ Open

Determining the distance patterns in the movements of future doctors in UK between 2002 and 2015: a retrospective cohort study

Journal:	*BMJ Open*
Manuscript ID	bmjopen-2023-077635.R2
Article Type:	Original research
Date Submitted by the Author:	13-Feb-2024

Complete List of Authors:	Hitchings, Lucy; Lancaster University<br>Fleet, Ben; Lancaster University, Lancaster Medical School<br>Smith, Daniel; General Medical Council<br>Read, Jonathan; Lancaster University Faculty of Health and Medicine, Centre for Health Informatics, Computing and Statistics, Lancaster Medical School<br>Melville, Colin; The University of Manchester; General Medical Council Manchester<br>Sedda , Luigi ; Lancaster University, Lancaster Ecology and Epidemiology Group, Lancaster Medical School

**Primary Subject Heading**:	Medical education and training
Secondary Subject Heading:	Health policy, Medical management
Keywords:	HEALTH SERVICES ADMINISTRATION & MANAGEMENT, EDUCATION & TRAINING (see Medical Education & Training), STATISTICS & RESEARCH METHODS

SCHOLARONE™
Manuscripts

I, the Submitting Author has the right to grant and does grant on behalf of all authors of the Work (as defined in the below author licence), an exclusive licence and/or a non-exclusive licence for contributions from authors who are: i) UK Crown employees; ii) where BMJ has agreed a CC-BY licence shall apply, and/or iii) in accordance with the terms applicable for US Federal Government officers or employees acting as part of their official duties; on a worldwide, perpetual, irrevocable, royalty-free basis to BMJ Publishing Group Ltd ("BMJ") its licensees and where the relevant Journal is co-owned by BMJ to the co-owners of the Journal, to publish the Work in this journal and any other BMJ products and to exploit all rights, as set out in our licence.

The Submitting Author accepts and understands that any supply made under these terms is made by BMJ to the Submitting Author unless you are acting as an employee on behalf of your employer or a postgraduate student of an affiliated institution which is paying any applicable article publishing charge ("APC") for Open Access articles. Where the Submitting Author wishes to make the Work available on an Open Access basis (and intends to pay the relevant APC), the terms of reuse of such Open Access shall be governed by a Creative Commons licence – details of these licences and which Creative Commons licence will apply to this Work are set out in our licence referred to above.

Other than as permitted in any relevant BMJ Author's Self Archiving Policies, I confirm this Work has not been accepted for publication elsewhere, is not being considered for publication elsewhere and does not duplicate material already published. I confirm all authors consent to publication of this Work and authorise the granting of this licence.

For peer review only

Determining the distance patterns in the movements of future doctors in UK between 2002 and 2015: a retrospective cohort study

Lucy A. Hitchings[1,#], Benjamin G. Fleet[2,#], Daniel Smith[3,&], Jonathan Read[4,&], Colin Melville[3,5,&], Luigi Sedda[6,&,*]

[1] Mathematics and Statistics, Lancaster University, Lancaster, LA1 4YG, UK.

[2] Lancaster Medical School, Lancaster University, Lancaster, LA1 4YG, UK.

[3] General Medical Council, Manchester, M3 3AW, UK.

[4] Centre for Health Information Computing and Statistics (CHICAS), Lancaster University, Lancaster, LA1 4YG, UK.

[5] School of Medical Sciences, Manchester University, M13 9PL, UK

[6] Lancaster Ecology and Epidemiology Group (LEEG), Lancaster University, Lancaster, LA1 4YG, UK.

[&] These authors jointly supervised this work.

* Correspondent author: l.sedda@lancaster.ac.uk

[#] Shared first authors.

For peer review only - http://bmjopen.bmj.com/site/about/guidelines.xhtml

Abstract

Objective To determine and identify distance patterns in the movements of medical students and junior doctors between their training locations.

Design A retrospective cohort study of UK medical students from 2002 to 2015.

Setting All UK medical schools, foundation and specialty training organisation.

Participants All UK medical students from 2002 to 2015, for a total of 97,932 participants.

Outcome measures Individual movements and number of movements by county of students from family home to medical school training, from medical school to foundation training and from foundation to specialty training.

Methods Leslie matrix, principal components analysis, Gini coefficient, chi-square test, generalised linear models and variable selection methods were employed to explore the different facets of students and junior doctors' movements from family home to medical school and for the full pathway.

Results The majority of the movements between the different training stages were restricted to a distance of up to 50 km; although the proportion of movements changed from year to year, with longer movements during 2007-2008. At the individual level, ethnicity, socio-economic class of the parent(s), and the deprivation score of the family home region were found to be the most important factors associated with the length of the movements from family home to medical school. Similar results were found when movements were aggregated at the county level, with the addition of factors such as gender and qualification at entry (to medical school) being statistically associated with the number of students moving between counties.

Conclusion Our findings show that while the future doctors do not move far from their family home or training location, this pattern is not homogeneous over time. Distances are influenced by demographic, socio-economic status and deprivation. These results may contribute in designing interventions aimed at solving the chronic problems of maldistribution and underdoctoring in UK.

Strengths and limitations of this study

For peer review only - http://bmjopen.bmj.com/site/about/guidelines.xhtml

- Use of a suite of statistical tools instead of isolated tests to ascertain significant movements scale and explanatory factors;
- first use of UKMED full data from 2002 to 2015
- full pathway available for only a small proportion of doctors
- additional socio-economic explanatory factors and policy interventions will need to be considered.

For peer review only

For peer review only - http://bmjopen.bmj.com/site/about/guidelines.xhtml

Introduction

The UK National Health Service (NHS) with its 1.5 million employees in England (1), 160,000 in Scotland (2), 106,000 in Wales (3), and with the Health and Social Care 73,000 employees in Northern Ireland (4), is the fifth largest employer in the world (5). The ratio between number of doctors (GPs and specialist) and served population is approximately 2 per 1,000 population. The process required to become a doctor or GP is lengthy and includes multiple training stages: Medical School, (4-6 years); Foundation training (2 years); and Specialty training (3-8 years) (6). The distribution of the doctors to population served in the UK is unequal (as shown for GPs in the past (7)), despite the sustained increase in doctor numbers in the last few years (1). However, the shortage of doctors is unlikely to be solved in the short term due to the structural and demographic characteristics of the underdoctored locations (8). Therefore, before any intervention is taken it is essential to understand the patterns in geographical movements of doctors and which factors are behind them. In fact, the potential maldistribution of doctors in the United Kingdom is contingent upon the spatial mobility of medical professionals, specifically in terms of their divergence from their geographic origins or training locations. If doctors predominantly remain within close proximity to their hometowns or training institutions, the resultant geographic distribution of healthcare providers is likely to mirror the demographic characteristics of medical school entrants and the geographic locales of these educational institutions and postgraduate training facilities. This, in turn, could deviate from an ideal distribution aligned with the broader healthcare needs of the population.

Previous research had provided partial answers to the questions regarding the patterns of movements of future doctors due to limitations in the data, methods and coarse spatial resolution. So far, analyses of these patterns has been descriptive and hardly statistically assessed, therefore figures remain crude and not comparable.

In a study dating back to 1997, Parkhouse et al. (9) found that only 38.2% of doctors stayed within the region of their family home, while 42% of individuals stayed in the same area as their medical school. However, at that time, London had a larger number of medical schools compared to the rest of UK. Hann et al. reported that between the mid 1980s and 2003, the maldistribution of GPs in England and Wales increased (7). This reduced between 1974 and 1994, although increased again in later years and by 2006 it was greater than the 1974 levels (10).

While previous research focused on the use of simple cross-tabulation (9), the Gini coefficient (11), and generalised linear models (12); they were often not sufficient to disentangle the complexity of the movements of doctors. To understand the spatial scales and explanatory factors associated with doctors' movements better, in addition to the traditional statistical analyses listed above, we have also employed a

For peer review only

new method, the Leslie matrix (usually applied in biology). We will show that our methods provide identification of the modal movement distances (short to long), their differences over time and the demographic and socio-economic factors associated with these scales.

Data and Methods

The data was provided from the UK Medical Education Database (UKMED) (project number UKMEDP44) and included 97,932 individuals who started medical school between 2002 and 2015. Of these, 871 had completed medical school and foundation training, and started or completed specialty training by the end of the study period (Supplementary File: Supplementary Information 1 and 2). We use the terms 'students' or 'medical students' to refer to those attending a medical school, and 'junior doctors' for those in foundation or specialty training. Since the dataset is only available up to 2015, the individuals for whom we have information for all three training stages are those who commenced medical school between 2002 and 2009. For all 97,932 students, the distances from the family home to medical school are available. Only individuals whose nationality was English, Welsh, Scottish or Northern Irish and whose medical degree was awarded from a UK university were considered in this study. The distances between the family home and the medical school considered only the medical school student's first choice, and therefore, subsequent changes are excluded. Out of 97,932 students, 5,093 (5.2%) changed medical schools. The 871 individuals include only those for whom the full training pathway was available without changes. 45 additional junior doctors who, at some point, were not in training or changed location during the foundation and specialty training were excluded. The analyses included 36 medical schools and all the hospitals providing training to the doctors across the UK.

This data was the full standardised data available at UKMED at that time. For each individual, the distances between each career stage (family home to medical school, medical school to foundation, foundation to specialisation) and other variables were provided (Table 1). For each individual, the distances were not summed (i.e., from the family home up to specialisation) and neither calculated between non-subsequent career stages (i.e., from the family home to foundation or specialisation or from medical school to specialisation). We used the term 'ethnicity' as per UKMED database, although there are on-going debates about its appropriate use (13) in particular for medical studies (14).

Six methods were used to investigate the movements of medical students across the UK. Four of these methods were integrated to investigate the movement distances and their patterns across the three stages and years: the Leslie model (known as a Leslie matrix), principal components analysis (PCA), Gini coefficient and chi-square test. Generalised linear models (GLMs) and variable selection methods were employed to evaluate the factors influencing the movements from family home to medical school (which

For peer review only - http://bmjopen.bmj.com/site/about/guidelines.xhtml

provide the largest data) at both individual and geographically aggregated levels. In practice, the first four analyses (Leslie, Gini, PCA and chi-square test) were applied to the 871 records containing the full pathways; while the Poisson GLMs and variable selection methods were applied on the whole 97,932 movements from family home to medical school.

The Leslie matrix is mostly applied to biological and ecological problems (15). The method is used to model population changes over time, including migration (16). It is a symmetric matrix with positive entries, where matrix cells contain the number of individuals passing from one stage to another. In this context, the stages are the education stages (medical school, foundation, specialisation). The matrix has been nested with distances, therefore for each stage, the number of students moving from one stage to the next is disaggregated by distance categories (17) (full method described in the Supplementary File: Supplementary Information 3). Since time is not integrated with space, the Leslie matrix is a simplified stage-only version. The lag distance applied is 25 km (0-25 km, 25-50 km, etc…). This lag allows for the identification of patterns at finer scales not possible if using a larger lag. Only the 871 individuals who completed all three training stages, were considered in the Leslie matrix construction.

The simplified stage-only Leslie matrix was analysed by employing a principal components analysis to evaluate the most important movements' distance categories among all training stages. This approach has been proposed by Usher and Williamson (17) and the resulting eigenvectors can be compared through chi-square tests to evaluate similarities and dissimilarities in the most important movements over years (18). In other words, annual Leslie matrices were created, and the relative first principal components (containing the largest amount of explained variance) for each year were extracted and compared in pairs by chi-square test.

To confirm equality or inequality in medical students and junior doctors (871 individuals) movement distance categories, the Gini coefficient has been calculated which has been used in similar researches (11). The Gini coefficient varies between 0 and 1 where 0 implies complete equality, and 1 complete inequality. The coefficient is derived from the Lorenz curve. When the Gini coefficient is 0, the Lorenz curve follows the line of equality, which is a straight diagonal line. If the distribution exhibits any inequality, the Lorenz curve falls below the line of equality. The Gini coefficient is the ratio between the Lorenz curve and the equality line. The Lorenz curve is the line passing through the points of cumulative percentage of the population (on the x-axis) and cumulative percentage of distances (on the y-axis). Finally, a Poisson generalised linear model was applied individually on the distance (outcome) covered by each individual student from family home to medical school (therefore on the full 97,932 students). Distance was assumed a count data with discrete and non-negative values. The model explanatory factors were gender, ethnicity, and various variables representing the socio-economic status of the students and their parents (full list presented in Table 1). In addition, in the second Poisson generalised linear model,

For peer review only

161 the number of students moving from the county of family home to the county of medical school was used as outcome. Since the data is aggregated by county, the model included the distance between training and family home counties and the modal values of each explanatory variable for both the departing (family home) and arriving (training) county.

Before running the individual and county-level multivariable Poisson generalised linear models, selection of the explanatory variables was carried out by univariate modelling and stepwise selection (19) since all the explanatory variables are categorical. The variable selection approach included the following steps:

1) fit univariate models with each potential exploratory variable (Table 1);
2) on each univariate model a log-likelihood ratio test (comparing with the null model) is performed using the function poisson.anova (20) in R-4.1.0software ;
3) the explanatory variable of the univariate model was included in the multivariable model if it caused the univariate model to be significantly different (at a significant level of 0.05) from the null model;
4) the multivariable model was then inputted in a stepwise selection method (function stepAIC in R-4.1.0 software(21)), with both forward and backward selection; the returned model with the lowest Akaike Information Criteria (AIC) (22, 23) was selected.

AIC was also employed to compare the Poisson regression model with a negative binomial model, which can account for overdispersion. The former yielded a lower AIC (indicating a better fit) than the negative binomial model, and for this reason, only the Poisson regression results are shown. A post-hoc test, the Tukey-Kramer test, was employed to compare pairwise differences within the variable categories. The study was performed using Strengthening the Reporting of Observational studies in Epidemiology (STROBE) guidance (24).

Patient and Public involvement

Patients or the public were not involved in the design, or conduct, or reporting, or dissemination plans of our research.

For peer review only - http://bmjopen.bmj.com/site/about/guidelines.xhtml

189 Table 1: List of variables used in the analyses and their definitions. * Individual Student-related variables;
190 ** Individual student's parents-related variables. Source: UKMED data dictionary (25).

Variable	Description	Categories
Bursary*	Whether the individual is in The University Clinical Aptitude Test (UCAT) questionnaire Bursary table.	Yes<br>No
Distance*	Coded as the travel distance between centroids.	0 = from the county of family home to the county of the medical school; 1 = from home postcode to the medical school postcode; 2 = from medical school postcode to foundation training postcode; 3 = from foundation training postcode to specialty training postcode.
Domicile County*	The county of the students' family home.	
Ethnicity*	Level 1 and Level 2 classification of ethnic groups as in 2011 Census.	Full list in Supplementary File: Supplementary Information 4.
BME*	Black and Minority Ethnic (BME).	0 = White<br>1 = BME
Gender*	The sex of the student.	0 = Male<br>1 = Female
Medical School County*	The county the medical school is in.	
Qualification*	The highest qualification the student has on entry to medical school.	1 = no formal qualification/not known;<br>2 = level 2 and below;<br>3 = level 3 including A levels and higher.
School Type*	Type of school the student attended between the ages of 11 and 16.	1 = independent/Private Fee Paying School; 2 = grammar School (selective); 3 = academy/Secondary School; 4 = further Education College / Sixth Form College; 5 = other.
Socio-Economic Classification*	Socio-economic background of students aged 21 and over at the start of their course, or for students under 21 the socio-economic background of their parent, step-parent or guardian who earns the most is returned. It is based on occupation, and if the parent or guardian is retired or unemployed, this is based on their most recent occupation.	1 = higher managerial & professional occupations; 2 = lower managerial & professional occupations; 3 = intermediate occupation; 4 = small employers & own account workers; 5 = lower supervisory & technical occupations; 6 = semi-routine occupations; 7 = routine occupations;8 = never worked & long-term unemployed; 9 = not classified.
Deprivation**	The Index of Multiple Deprivation for England, Northern Ireland, Scotland and Wales.	1 = most deprived; 2; 3; 4; 5 = least deprived.
Income	Whether the student's home has had any	Yes

For peer review only

For peer review only - http://bmjopen.bmj.com/site/about/guidelines.xhtml

Support**	income support during their time at school.	No
Occupation**	Occupation of the household reference person. This is the person responsible for owning or renting or who is otherwise responsible for the accommodation. In the case of joint householders, the person with the highest income takes precedence. Where incomes are equal, the oldest person is taken as household reference. Based on the National Statistics Socio-Economic.	1 = managerial and professional occupations; 2 = intermediate occupations; 3 = small employers and account workers; 4 = lower supervisory and technical occupations; 5 = semi-routine and routine occupations.
Occupation individual**	Socioeconomic class occupation description of parent 1 and parent 2 from UCAT	1 = modern professional (e.g. teacher, nurse, social worker, artist, police officer (sergeant or above)); 2 = clerical & intermediate (e.g. secretary, call centre agent, nursing auxiliary, nursery nurse); 3 = senior managers or administrators (e.g. finance manager, chief executive); 4 = technical & craft (e.g. motor mechanic, plumber, printer, tool maker, gardener, train driver, fitter); 5 = semi-routine manual & service (e.g. postal / farm worker, security guard, catering/sales assistant); 6 = routine manual & service (e.g. HGV driver, cleaner, porter, sewing machinist, bar staff, labourer); 7 = middle or junior managers (e.g. office / retail / bank / restaurant / warehouse manager, publican); 8 = traditional professional (e.g. accountant, solicitor, medical practitioner, scientist, civil servant); 9 = never worked; 10 = don't know or prefer not to say.

Results

The mean and standard deviation of the distances between each training stage are shown in Table 2. The shorter, although more dispersed, distances are from the medical school to foundation programme training.

The principal components analysis of the simplified stage-only Leslie matrix, applied to only the individuals for which the full pathway is known (871 individuals), found that out of 40 principal components (one for each distance category), the first principal component (distances up to 25 km)

For peer review only - http://bmjopen.bmj.com/site/about/guidelines.xhtml

199 explained 41% of the variance in the movements' distances, the second 13% (25-50 km) and the third 8% (50-75 km) (Supplementary File: Supplementary Information 5). The highest proportion of individuals (54%) concentrates in the shorter movement distances (up to 50 km) than longer movement distances. These results have been confirmed by the value of the Gini coefficient for the Leslie matrix, which was 0.936 (Supplementary File: Supplementary Information 6) meaning inequality on the distribution of movements among all categories and stages, with modal distances at a short scale. These results indicate that most of the individuals stayed close to their home or training organisation.

Table 2. Summary statistics for the three career stage movements in the full dataset.

Distances	Mean (km)	Standard deviation (km)
From family home to medical school	173.8	155.6
From medical school to foundation training location	126.8	226.9
From foundation to specialty training location	161.8	216.8

The proportions of movements among the distance categories are heterogeneous in time. These differences have been tested by the chi-square test applied to paired first principal components of annual Leslie matrices (from 2002 to 2009 – where 2009 is the last year for which we have a full pathway for students). Figure 1 shows that 2002 proportion of movements are significantly different from most of the rest of the years (in red) at a significant level of 0.05, apart from 2007. 2004 was similar only to 2003 and 2009; 2006 to 2005, 2007 and 2009; and 2008 to 2005, 2007 and 2009. The year that was similar to most of all the other years is 2009, which was different only to 2002. Notably, the years with longest distances were 2007 and 2008 where the majority of individuals travelled distances up to 100-125 km (Supplementary File: Supplementary Information 5).

To evaluate determinants of the movement from family home to medical school at both individual and aggregated (by county) level, generalised linear models were employed within a variable selection framework, the latter aimed at selecting the model with the lowest AIC or in other words the best model fitting.

Table 3: Poisson generalised linear model results for individual movements. Occ: occupation of the household reference; REF: reference category.

Variable	Coefficient	Standard Error	*p-value*
Intercept	5.097	0.005	< 0.001
Occ. managerial	REF		
Occ. intermediate	-0.079	0.002	< 0.001

For peer review only - http://bmjopen.bmj.com/site/about/guidelines.xhtml

Occ. small employers	-0.061	0.002	< 0.001
Occ. lower supervisory	-0.089	0.003	< 0.001
Occ. semi-routine	-0.080	0.003	< 0.001
Deprivation (Level 1)	REF		
Deprivation (Level 2)	0.003	0.001	< 0.001
Deprivation (Level 3)	-0.045	0.001	< 0.001
Deprivation (Level 4)	-0.129	0.001	< 0.001
Deprivation (Level 5)	-0.273	0.001	< 0.001
British	REF		
African	-0.071	0.002	< 0.001
Arab	-0.375	0.003	< 0.001
Bangladeshi	-0.427	0.003	< 0.001
Caribbean	-0.190	0.005	< 0.001
Chinese	-0.088	0.002	< 0.001
Gypsy or Irish Traveler	0.074	0.040	0.061
Indian	-0.281	0.001	< 0.001
Irish	0.239	0.001	< 0.001
Pakistani	-0.356	0.001	< 0.001
White and Asian	-0.023	0.002	< 0.001
White and Black African	0.051	0.004	< 0.001
White and Black Caribbean	-0.055	0.004	< 0.001
Other Asian background	-0.328	0.001	< 0.001
Other Black background	-0.044	0.007	< 0.001
Other Mixed background	-0.095	0.002	< 0.001
Other White background	0.027	0.001	< 0.001
Other Ethnic group	-0.283	0.002	< 0.001
Not stated	-0.015	0.006	0.006

At individual level, the best fitting model of the distances travelled by students was obtained with ethnicity sub-group (Level 2 information), student's parents' occupation and deprivation (Table 3). This model is significantly different (in terms of reduction of residual deviance) from any model based on the three individual variables and their combinations (anova test). All the classes of deprivation and parent occupation were found to be statistically significant. In terms of deprivation, compared to the most

For peer review only - http://bmjopen.bmj.com/site/about/guidelines.xhtml

For peer review only

deprived, the least deprived levels (3 to 5) travelled a shorter distance when they moved from their family home to medical school. Compared to students with parents in managerial and professional occupation, the students with other occupations travelled shorter distances (Table 3). In terms of the ethnicity variable, most of the ethnic groups travelled shorter distances than the White British, apart from the Irish, White and Black African and Other White background (Gypsy or Irish traveller coefficient was not significantly different from zero).

Among students traveling shorter distances than those with White British background, a four to five-fold difference was observed between those of Bangladeshi, Arab, Pakistani, and other Asian backgrounds, compared to students of Chinese, Other Mixed and African backgrounds (travelling longer distances compared to them but shorter than White British), and six to ten-fold difference in comparison to students of White and Black Caribbean, and Other Black backgrounds. Moreover, there was a more than 15-fold difference in comparison to students of White and Asian backgrounds, as well as those whose background was not stated when compared to Bangladeshi, Arab, Pakistani, and other Asian backgrounds (Table 3).

Students of Irish background travelled the longest distances compared to those of White British background.

Table 4: Poisson generalised liner model results for county-aggregated data. Departing = county of family home; arriving = county with the medical school.

Variable	Estimate	Standard Error	*p-value*
Intercept	-0.963	0.068	< 0.001
Distance between counties	-0.006	0.000	< 0.001
BME departing county (No)	REF		
BME departing county (Yes)	-1.204	0.042	< 0.001
Gender departing county (Male)	REF		
Gender departing county (Female)	2.691	0.029	< 0.001
Qualification departing county (below level 3/not known)	REF		
Qualification departing county (level 3)	0.085	0.004	< 0.001
Deprivation departing county (level 1)	REF		
Deprivation departing county (above level 1)	-0.454	0.005	< 0.001
Deprivation arriving county (level 1)	REF		
Deprivation arriving county (above level 1)	-0.483	0.006	< 0.001
Students socio-economic (higher managerial)	REF		

For peer review only

For peer review only - http://bmjopen.bmj.com/site/about/guidelines.xhtml

Students socio-economic (lower and below)	-0.183	0.021	< 0.001

A multiple comparison test (Tukey-Kramer test) highlighted a pairwise significant difference between White British and: African, Bangladeshi, Caribbean, Chinese, Indian, Irish, White and Asian, White and Black African, White and Black Caribbean, Other Asian, Other Ethnic, and Other Mixed background (*p-value* < 0.001). Within the group Bangladeshi, Arab, Pakistani, and Other Asian backgrounds, only Bangladeshi – Arab comparison was found significantly different.

The best fitting model by county (Table 4) confirms the importance of deprivation in the number of students moving from family home to medical school counties. Distance between counties was an important factor for the number of students moving from one county to the other, with medical schools having more students coming from neighbouring counties than from counties further away (negative association with distance). The rest of the most significant variables were all associated with the departing county: BME, female students, highest qualification at entry, and SEC (Table 4). In particular, counties with higher proportions of BME or with higher deprivation or socio-economic status were associated with fewer individuals moving. While counties with a larger proportion of female students or higher qualification at entry were associated with larger number of movements.

Discussion

This research found that the largest proportion of individuals travelled within 25 km to the next stage of professional development, with most of the movements being statistically significant at less than 50 km, although not for every year under study. In fact, in the years of the financial crisis (2007-2008) (26) individuals within the three training stages travelled further (up to 125 km). Although this study was not aimed at investigating the effect of the financial crisis on UK medical students' movements, it is certainly the first study proposing this hypothesis. A study on medical students in Spain reported that students focused more on career prospects than lifestyle in response of the economic crisis (27), when choosing their specialty. If this was the reason for longer movements by UK medical students and junior doctors it is something that will need to be ascertained.

The up-to 50 km modal distance travelled by UK-born medical students and junior doctors for all the years between 2002 and 2009 is reported here for the first time. A 2019 GMC report that focused on doctors who gained their UK Primary Medical Qualification between 2012 and 2018 found that almost a quarter (23%) of doctors in 2019 were working within 10 miles (16 km) of where they studied at medical school (28). However, a third of graduates moved more than a hundred miles.

For peer review only

284 Despite the absence of distance information, a previous study (on a restricted number of doctors and obtained by questionnaire) reported that, between 1974 and 2008, 36% of the doctors stayed in the same region as their family home during medical school and 48% undertook specialist training in the same region as their medical school (29). A more recent observational study that focused on doctors graduating between 2012 and 2014, found that the majority of doctors trained in foundation schools that were close to their family homes (30).

290 We found strong inequality in the movements – e.g. the presence of dominant movement distance categories compared to others. A previous study using Gini coefficient applied to movements for general practitioners between 1974 and 2003 (7) found greater equality (Gini coefficient between 0.4 and 0.5) than the present research. A direct comparison between the two studies cannot be done, but the increase in medical student number supply since 1974 (7) could have caused a more pronounced movements inequality (as we found) than previously.

296 The main factors associated with students' movement distances and student counts from family home to medical school are very similar at both individual and aggregated – by county – levels. The most important factors were socioeconomic status, deprivation score and ethnicity (BME, Ethnicity group and sub-groups). In our analyses, increased deprivation was found to be associated with both fewer doctors moving and shorter distances moved, while more students with higher degrees at entry to medical school or with parents with professional occupation tended to move further away. In previous literature, Primary Care Trusts (PCT) in England were seen to be attractive if there is less crime in the area, or better schools and opportunities (10). Similarly those students with parents in NSSEC1 (professional occupations) were less likely to work in deprived areas compared to those in NSSEC5 (routine occupations) (31). Regarding the ethnicity, one study reported that doctors from non-white ethnic backgrounds were more likely to train at foundation schools closer to their family home (30). We found the same findings but in reference to medical schools although our analyses do not account for the potential geographical clustering of ethnic minorities in the UK (32) which can potentially bias the results. Personal factors such as ethnicity, education and gender were all significant contributing factors to the movement of doctors at both individual and county levels (33).

311 Consideration of the structural elements of our societies, such as deprivation and demography (e.g. ethnicity), will require different type, spatial scale and economic effort for improving the distribution of doctors in the UK. The NHS proposed a 'rural weighting' payment in England which would aim to attract doctors and GPs to rural areas with a financial incentive (34, 35). This may be equally applicable in areas of high deprivation. Payments of this type could also be increased in line with the length of service thereby increasing retention of medical practitioners within rural or deprived areas. Another potential solution is expanding widening access to medical schools and create new medical schools in areas

For peer review only

For peer review only - http://bmjopen.bmj.com/site/about/guidelines.xhtml

underserved given the tendency of the students to stay local to their training institution (36). In Scotland it has been found that there is a link between childhood background, as defined by parental socioeconomic status and being in more remote residence, and the population GPs are likely to serve(31). The authors suggested that GPs residing in more rural areas and those who experienced greater deprivation during their childhood tended to work in settings that reflected these characteristics. While this should not be presumed from these students, World Health Organisation recommendations emphasise the need to create more opportunities, such as locating educational facilities closer to rural areas, and providing incentives, such as benefits or direct payments, to medical students and doctors to serve in rural areas (37). However, recent literature indicates that outcomes of financial incentives are limited or unclear (38). Family-unit and community considerations are often the predominant factors influencing doctors' movements (39).

The main limitation of our study is the absence of historical mapping for all distances between medical schools, foundation, and specialty training facilities. Currently, this information is unavailable, but it is crucial to test alternative hypotheses regarding the likelihood of movement distance in relation to the availability of training facilities in the surrounding area, weighted by the number of positions available at the training location.

Other limitations are also present. This study includes information up to 2015, which may not reflect recent changes in medical education or workforce dynamics. Students who left their medical education or moved between training locations are not included in the dataset; therefore, the results are not generalisable for this group of students. We use the term 'ethnicity' as per the UKMED database, based on the categorisation adopted by the Office for National Statistics. However, this may not be appropriate due to limitations in the way this variable is defined and measured.

Finally, the present study did not consider international medical graduates working in the UK, UK-born doctors working abroad, or records pre-2002. Data for these doctors were not available. A new study (NIHR134540) (40) is investigating why doctors work where they work and how this is influenced by the organisation of the healthcare system, the social and physical environment where the future doctors grow up and move as well as their individual beliefs and choices. This new study which uses mixed methods and a larger dataset than any previously should increase the understanding around doctors movements from the doctor's perspectives as well and not only from data records. We hope that our study and the ones currently active will identify the best interventions to improve the underdoctoring and maldistribution of doctors across the UK.

Author Contributions:

CM and LS conceived and designed the study. LH, DS, JR CM and LS discussed and drafted the

For peer review only - http://bmjopen.bmj.com/site/about/guidelines.xhtml

study protocol. LH and BF screened and selected the articles. LH and BF analysed the data. LH, BF and LS interpreted the results. LH, BF and LS drafted the manuscript. All authors critically revised the manuscript, contributed to revising the manuscript and had final responsibility for the decision to submit for publication. DS, JR, CM and LS supervised the study. The corresponding author attests that all listed authors meet authorship criteria and that no others meeting the criteria have been omitted.

Competing interests:

CM is medical director and director of education and standards at the General Medical Council. Views expressed in this article do not necessarily reflect those of the General Medical Council.

Data sharing

The data for this research was accessed through the cloud from the UK Medical Education Database (UKMED), specifically from the UKMEDP044 extract generated on 01/06/2018. The availability of the data used in this study is through an application process to UKMED, and more information can be found on their website: https://www.ukmed.ac.uk/. Approved for publication on 30/06/2023.

Funding

LS is supported by the NIHR Health and Social Care Delivery Research (HSDR) programme (NIHR134540) and North West Cancer Research UK (LI2021SEDDA). The views expressed are those of the author(s) and not necessarily those of the NIHR or the Department of Health and Social Care.

Ethical approval

The Medical Schools Council agreed that all approved applications for research projects using data exclusively held by UKMED would meet the criteria for a blanket exemption from the need to apply for ethics approval that would be recognised by ethics committees relevant to the UK medical schools. A letter was written from QMUL Ethics of Research Committee on behalf of all UK medical schools to confirm ethics exemption (available at chrome-extension://efaidnbmnnnibpcajpcglclefindmkaj/https://www.ukmed.ac.uk/documents/UKMED_research_projects_ethics_exemption.pdf).

Acknowledgments

We are grateful to UKMED for the use of these data. However, UKMED has no responsibility for the

For peer review only

For peer review only - http://bmjopen.bmj.com/site/about/guidelines.xhtml

analysis or interpretation of their data. The data includes information derived from that collected by the Higher Education Statistics Agency Limited ("HESA") and provided to the GMC ("HESA Data"). Source: HESA Student Records 2002/2015 Copyright Higher Education Statistics Agency Limited. The Higher Education Statistics Agency Limited makes no warranty as to the accuracy of the HESA Data, cannot accept responsibility for any inferences or conclusions derived by third parties from data or other information supplied by it.

References

1. Rolewicz L, Palmer B, Lobont C. The NHS workforce in numbers: Nuffield Trust; 2022 [Available from: https://www.nuffieldtrust.org.uk/resource/the-nhs-workforce-in-numbers.
2. Government S. Health Workforce [Available from: https://www.gov.scot/policies/health-workforce/.
3. Government W. Staff directly employed by the NHS: as at 30 September 2022 2022 [Available from: https://www.gov.wales/staff-directly-employed-nhs-30-september-2022-html.
4. Health Do. Northern Ireland health and social care (HSC) workforce statistics December 2022 2023 [Available from: https://www.health-ni.gov.uk/publications/northern-ireland-health-and-social-care-hsc-workforce-statistics-december-2022.
5. McCarthy N. The World's Biggest Employers Jersey City, NJ, US: Forbes; 2015 [Available from: https://www.forbes.com/sites/niallmccarthy/2015/06/23/the-worlds-biggest-employers-infographic.
6. Brice J, Corrigan O. The changing landscape of medical education in the uk. . Medical Teacher. 2010;32(9):727-32.
7. Hann M, Gravelle H. The maldistribution of general practitioners in england and wales: 1974-2003. The British journal of general practice : the journal of the Royal College of General Practitioners. 2004;54(509).
8. British Medical Association. NHS medical staffing data analysis2023 06/07/2023. Available from: https://www.bma.org.uk/advice-and-support/nhs-delivery-and-workforce/workforce/nhs-medical-staffing-data-analysis.
9. Parkhouse J, Lambert T. Home, training and work: Mobility of british doctors. 1997;31:399-407.
10. Goddard M, Gravelle H, Hole A, Marini G. Where did all the gps go? increasing supply and geographical equity in england and scotland. Journal of health services research & policy,. 2010;15(1):28-35.
11. Morita T, Tanimoto T, Morita M, Tsubokura M, M. K. Trend in unequal geo-graphical distribution of doctors by age and sex in japan from 2004 to 2014. Public Health. 2018;159:95-8.
12. Ide H, Doi S, Atarashi H, Fujita S, Koike S. The distance and chance of lifetime geographical movement of physicians in japan: an analysis using the age-period-cohort model. Human Resources for Health. 2018;16(1).
13. Flanagin A, Frey T, Christiansen SL. Updated Guidance on the Reporting of Race

For peer review only

For peer review only - http://bmjopen.bmj.com/site/about/guidelines.xhtml

and Ethnicity in Medical and Science Journals. Jama-J Am Med Assoc. 2021;326(7):621-7.
14. Martinez RAM, Andrabi N, Goodwin AN, Wilbur RE, Smith NR, Zivich PN. Conceptualization, Operationalization, and Utilization of Race and Ethnicity in Major Epidemiology Journals, 1995-2018: A Systematic Review. Am J Epidemiol. 2023;192(3):483-96.
15. Monte L. Characterisation of a nonlinear leslie matrix model for predicting the dy- namics of biological populations in polluted environments: Applications to radioecology. Ecological Modelling. 2013;248:174-83.
16. Leslie P. On the use of matrices in certain population mathematics. 1945;33:183-212.
17. Usher M, Williamson M. A deterministic matrix model for handling the birth, death, and migration processes of spatially distributed populations. . 1970;26:1-12.
18. Freund R. Symmetric matrices and eigendecomposition2014. Available from: http://s3.amazonaws.com/mitsloan-php/wp-faculty/sites/30/2016/12/15032137/Symmetric-Matrices-and-Eigendecomposition.pdf.
19. Upton G, Cook I. Forward selection 2014 [Available from: http://www.oxfordreference.com/view/10.1093/acref/9780199679188.001.0001/acref-9780199679188-e-624.
20. Tsagris M, Papadakis M. Taking R to its limits: 70+ tips. PeerJ Preprints. 2018;6.
21. Ripley BD. Modern applied statistics with S: springer; 2002.
22. Symonds M, Moussalli A. A brief guide to model selection, multimodel inference and model averaging in behavioural ecology using akaike's information criterion. Behavioral Ecology and Sociobiology. 2011;65(1):13-21.
23. Gelman A. Discussion paper: Analysis of variance - why it is more important than ever. The Annals of Statistics. 2005;33(1):1-33.
24. Vandenbroucke JP, von Elm E, Altman DG, Gotzsche PC, Mulrow CD, Pocock SJ, et al. Strengthening the Reporting of Observational Studies in Epidemiology (STROBE): explanation and elaboration. Epidemiology. 2007;18(6):805-35.
25. UKMED - 2018 V1. London: UKMED; 2018. Data Dictionary.
26. Lindstrom M, Giordano GN. The 2008 financial crisis: Changes in social capital and its association with psychological wellbeing in the United Kingdom - A panel study. Soc Sci Med. 2016;153:71-80.
27. Harris JE, Lopez-Valcarcel BG, Ortun V, Barber P. Specialty choice in times of economic crisis: a cross-sectional survey of Spanish medical students. Bmj Open. 2013;3(2).
28. GMC. The state of medical education and practice in the UK 2019 [Available from: https://www.gmc-uk.org/-/media/documents/the-state-of-medical-education-and-practice-in-the-uk---workforce-report_pdf-80449007.pdf.
29. Goldacre M, Davidson J, Maisonneuve J, Lambert T. Geographical movement of doctors from education to training and eventual career post: UK cohort studies. J R Soc Med. 2013;106(3):96-104.
30. Kumwenda B, Cleland JA, Prescott GJ, Walker KA, Johnston PW. Geographical mobility of UK trainee doctors, from family home to first job: a national cohort study. BMC Med Educ. 2018;18(1):314.
31. Dowell J, Norbury M, Steven K, Guthrie B. Widening access to medicine may improve general practitioner recruitment in deprived and rural communities: survey of

For peer review only - http://bmjopen.bmj.com/site/about/guidelines.xhtml

gp origins and current place of work. BMC Medical Education. 2015;15(1).
32. Shaw B, Menzies L, Bernardes E, Baars S, Nye P, Allen R. Ethnicity, gender and social mobility. London: Social Mobility Commission. 2016.
33. Dussault G, Franceschini M. Not enough there, too many here: understanding geographical imbalances in the distribution of the health workforce. Human Resources for Health. 2006.
34. RSN RSN. Shortage of rural doctors puts patients' health at risk. 2017 [Available from: http://rsnonline.org.uk/shortage-of-rural-doctors-puts-patients-health-at-risk.
35. Rolewicz L, Palmer B. Placed at a premium? The use of recruitment and retention pay supplements to address staffing shortfalls: Nuffield Trust; 2022 [Available from: https://www.nuffieldtrust.org.uk/research/placed-at-a-premium.
36. Verma P, Ford JA, Stuart A, Howe A, Everington S, Steel N. A systematic review of strategies to recruit and retain primary care doctors. BMC Health Serv Res. 2016;16:126.
37. WHO. WHO guideline on health workforce development, attraction, recruitment and retention in rural and remote areas. World Health Organization Geneva; 2021.
38. Swami M, Scott A. Impact of rural workforce incentives on access to GP services in underserved areas: Evidence from a natural experiment. Soc Sci Med. 2021;281:114045.
39. Holloway P, Bain-Donohue S, Moore M. Why do doctors work in rural areas in high-income countries? A qualitative systematic review of recruitment and retention. Aust J Rural Health. 2020;28(6):543-54.
40. NIHR. HSDR Funding Committee Public Minutes March 2022 2022 [Available from: https://www.nihr.ac.uk/documents/hsdr-funding-committee-public-minutes-march-2022/30467.

For peer review only - http://bmjopen.bmj.com/site/about/guidelines.xhtml

Figure Legend

Figure 1: Chi-square p-values from comparison of annual first components of the Leslie matrix. p-values below 0.05 are shown in red.

For peer review only

For peer review only - http://bmjopen.bmj.com/site/about/guidelines.xhtml

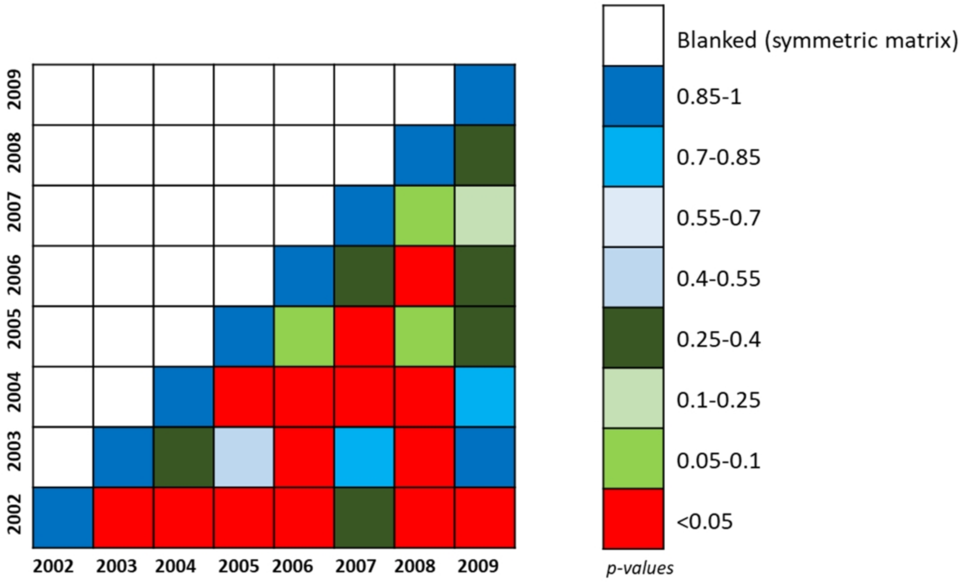

Figure 1. Chi-square p-values from comparison of annual first components of the Leslie matrix. p-values below 0.05 are shown in red.

1200x800mm (96 x 96 DPI)

Determining the distance patterns in the movements of future doctors in UK between 2002 and 2015: a retrospective cohort study

Lucy A. Hitchings, Benjamin G. Fleet, Daniel Smith, Jonathan Read, Colin Melville, Luigi Sedda

Supplementary File

Contents

Supplementary information 1: Table S1. Cohort of medical students and junior doctors considered int the study. Foundation year one (F1) and Specialty year one (S1) numbers are referred to individuals that started the medical school between 2002 and 2015. 2

Supplementary information 2: Table S2. Number of junior doctors starting S1 between 2011 and 2015 (second row) and contribution by medical school year. 3

Supplementary information 3: A simplified stage only Leslie Matrix 4

Supplementary information 4: UKMED Ethnicity classification 5

Supplementary information 5: Principal component analysis results 6

Supplementary information 6: Lorenz curve 8

For peer review only

Supplementary information 1: Table S1. Cohort of medical students and junior doctors considered int the study. Foundation year one (F1) and Specialty year one (S1) numbers are referred to individuals that started the medical school between 2002 and 2015.

Year	Number medical students starting medical school	Number of junior doctors in F1	Number of junior doctors in S1
2002	6640	0	0
2003	6824	0	0
2004	7190	0	0
2005	7201	0	0
2006	7145	0	0
2007	7042	0	0
2008	7193	0	0
2009	7100	4487	0
2010	7130	5331	0
2011	7193	5684	137
2012	6936	6494	309
2013	6751	6378	236
2014	6810	6752	114
2015	6777	6597	75
Totals	*97932*	*41723*	*871*

For peer review only

Supplementary information 2: Table S2. Number of junior doctors starting S1 between 2011 and 2015 (second row) and contribution by medical school year.

Year junior doctors started S1	2011	2012	2013	2014	2015
Number of junior doctors in S1	137	309	236	114	75
Proportion (in %) of junior doctors in S1 (second row) that started medical school in 2002	14.39	3.39	2.65	2.75	2.78
Proportion (in %) of junior doctors in S1 (second row) that started medical school in 2003	75.76	29.49	11.95	14.68	6.94
Proportion (in %) of junior doctors in S1 (second row) that started medical school in 2004	6.82	58.98	18.14	28.44	8.33
Proportion (in %) of junior doctors in S1 (second row) that started medical school in 2005	2.27	7.12	63.72	26.61	31.94
Proportion (in %) of junior doctors in S1 (second row) that started medical school in 2006	0.76	0.68	2.21	23.85	20.83
Proportion (in %) of junior doctors in S1 (second row) that started medical school in 2007	0.00	0.34	1.33	0.00	22.22
Proportion (in %) of junior doctors in S1 (second row) that started medical school in 2008	0.00	0.00	0.00	0.92	3.39
Proportion (in %) of junior doctors in S1 (second row) that started medical school in 2009	0.00	0.00	0.00	2.75	3.57

For peer review only

Supplementary information 3: A simplified stage only Leslie Matrix

A distance matrix is a square matrix that consists of the distances between elements, where the elements are the three stages required to complete the medical career. Those three stages are medical school, foundation training and specialty training. Movements are defined as the distance covered by the students from and to postcodes of two successive stages. Movement distances are lagged at 25 km interval.

The following example describes the construction of the Leslie matrix for the first three distances (D1=0-25km, D2=25-50km and D3=50-75km). First consider students travelling from family home to medical school, they are grouped by distance along the matrix diagonal (matrix A):

$$A = \begin{bmatrix} D_1 & & \\ & D_2 & \\ & & D_3 \end{bmatrix}$$

For each of the students who travelled a particular distance onto medical school, each of these will also have moved a certain distance onto their respective foundation training. Starting from the students in D1, they are split between students that travelled 0-25 km (F1), 25-50 km (F2) and 50-75 km (F3). In the same way for students in D2 and D3 (matrices B).

$$B(D_1) = \begin{bmatrix} F_{1,1_{D_1}} & F_{1,2_{D_1}} & F_{1,3_{D,1}} \\ F_{2,1_{D_1}} & & \\ F_{3,1_{D_1}} & & \end{bmatrix}, \quad B(D_2) = \begin{bmatrix} & F_{1,2_{D_2}} & \\ F_{2,1_{D_2}} & F_{2,2_{D_2}} & F_{2,3_{D_2}} \\ & F_{3,2_{D_2}} & \end{bmatrix},$$

$$B(D_3) = \begin{bmatrix} & & F_{1,3_{D_3}} \\ & & F_{2,3_{D_3}} \\ F_{3,1_{D_3}} & F_{3,2_{D_3}} & F_{3,3_{D_3}} \end{bmatrix}$$

Since the matrices are symmetric, $F_{1,2}$ and $F_{2,1}$ have the same values. The same matrices can be created for the F1 distance students going to specialty training (C matrices):

$$C(F_{1,1}) = \begin{bmatrix} S_{1,1_{F1,1}} & S_{1,2_{F1,1}} & S_{1,3_{F1,1}} \\ S_{2,1_{F1,1}} & & \\ S_{3,1_{F1,1}} & & \end{bmatrix}$$

Where $S_{1,1}$ is the number of students that travelled 0-25km in all the stages, i.e. from home to medical school (D1), from medical school to foundation (F1) and from foundation to specialty training (S1). Finally, the Leslie matrix is the sum of matrix A with matrices B and C. This matrix is a symmetric matrix with positive entries and has the same structure as a Leslie matrix, although a Leslie matrix is formulated based on divisions such as stage and age, assuming only population flows in adjacent cells of the matrix. By considering only non-overlapping stages (Murray and Sandercock, 2020), flow to non-contiguous cells is possible (Usher and Williamson, 1970).

References

Murray, Dennis L., and Brett K. Sandercock, eds. Population ecology in practice. John Wiley & Sons, 2020.

Usher, M. B., and M. H. Williamson. "A deterministic matrix model for handling the birth, death, and migration processes of spatially distributed populations." Biometrics (1970): 1-12.

For peer review only

Supplementary information 4: UKMED Ethnicity classification

Level 1 (bold) and Level 2 Ethnicity.
https://www.ethnicity-facts-figures.service.gov.uk/style-guide/ethnic-groups

Asian or Asian British

- Indian
- Pakistani
- Bangladeshi
- Chinese
- Any other Asian background

Black, African, Caribbean or Black British

- African
- Caribbean
- Any other Black, African or Caribbean background

Mixed or multiple ethnic groups

- White and Black Caribbean
- White and Black African
- White and Asian
- Any other Mixed or multiple ethnic background

White

- British (English, Welsh, Scottish, Northern Irish)
- Irish
- Gypsy or Irish Traveller
- Any other White background

Other ethnic group

- Arab
- Any other ethnic group

For peer review only

Supplementary information 5: Principal component analysis results

Table S3: Proportion of variance explained by the distance categories for all years and by each year from principal component analysis applied to all years and yearly Leslie matrices respectively.

km	Overall	2002	2003	2004	2005	2006	2007	2008	2009
0-25	40.98%	42.70%	40.07%	43.12%	45.34%	31.28%	28.55%	28.19%	43.45%
25-50	13.19%	1.32%	12.17%	15.84%	15.87%	10.82%	5.58%	4.72%	14.91%
50-75	7.84%	5.27%	6.47%	9.07%	9.83%	2.62%	6.01%	0%	0%
75-100	4.75%	5.09%	4.98%	5.25%	2.94%	8.12%	7.48%	6.17%	0%
100-125	8.47%	11.15%	7.34%	7.73%	8.36%	6.91%	12.70%	21.80%	0%
125-150	6.00%	9.83%	7.06%	3.07%	3.78%	15.86%	14.69%	7.72%	10.68%
150-175	1.11%	1.50%	1.29%	0.45%	1.23%	4.31%	1.11%	<0.1%	0%
175-200	1.35%	2.39%	1.61%	1.24%	0.37%	0.76%	2.53%	1.54%	6.69%
200-225	0.74%	0%	0.72%	0.57%	0.28%	2.01%	0.49%	4.64%	0%
225-250	1.17%	5.95%	2.06%	1.62%	0.64%	1.62%	0%	1.46%	4.59%
250-275	0.51%	2.26%	0.77%	0.21%	0.23%	0.51%	1.11%	1.13%	1.81%
275-300	0.36%	0%	0.72%	<0.1%	0.59%	0%	0%	0%	0%
300-325	1.34%	0.17%	3.97%	0.22%	0.63%	0.14%	1.15%	0.23%	0%
325-350	0.42%	1.20%	0.46%	0.40%	0.22%	<0.1%	1.11%	0.15%	0%
350-375	1.50%	1.36%	2.26%	1.69%	0.61%	3.88%	0%	3.08%	0%
375-400	0.78%	0%	1.24%	1.15%	0.43%	0%	2.18%	<0.1%	0%
400-425	0.56%	0%	1.01%	0.39%	0.41%	2.19%	<0.1%	1.07%	0%
425-450	0.58%	0%	0.44%	0.58%	0.78%	2.16%	0%	<0.1%	0%
450-475	4.43%	4.60%	2.32%	5.07%	4.20%	2.42%	5.66%	1.93%	0%
475-500	0.36%	1.19%	0.51%	0.26%	0.30%	1.01%	0%	1.48%	0%
500-525	0.33%	1.65%	0.27%	<0.1%	0.20%	0%	0%	1.47%	0%
525-550	<0.1%	0%	<0.1%	<0.1%	<0.1%	0.57%	1.07%	<0.1%	0%
550-575	0.27%	0%	0%	0%	0.20%	0.41%	4.04%	0.68%	0%
575-600	0.22%	0%	0.43%	<0.1%	0.14%	0.99%	0%	0%	0%
600-625	0.14%	0%	0%	0.21%	0.28%	0%	0%	0%	0%
625-650	<0.1%	0%	0%	0.18%	<0.1%	0%	1.08%	0%	0%
650-675	0.16%	0%	0.21%	<0.1%	0.20%	<0.1%	1.07%	0%	0%
675-700	0.11%	<0.1%	0%	<0.1%	0.20%	0%	0%	0%	7.90%
700-725	0.26%	1.13%	<0.1%	<0.1%	0.26%	0%	0.99%	1.39%	0%
725-750	0.42%	0%	0.65%	0.23%	0.41%	0%	1.10%	6.30%	0%
750-775	0.18%	0%	<0.1%	<0.1%	0.58%	<0.1%	0%	<0.1%	0%

For peer review only

775-800	0.71%	1.20%	0.44%	0.97%	<0.1%	<0.1%	0.23%	2.46%	0%
800-825	0.13%	0%	0%	0.19%	0.26%	0%	<0.1%	0%	0%
825-850	0.17%	0%	0.24%	0.18%	<0.1%	0%	0%	0%	0%
850-875	0.18%	0%	0%	0%	0.20%	0%	<0.1%	0%	9.97%
875-900	<0.1%	0%	<0.1%	0%	0%	1.34%	0%	2.15%	0%
900-925	<0.1%	<0.1%	0%	0%	0%	0%	0%	0%	0%
925-950	0%	0%	0%	0%	0%	0%	0%	0%	0%
950-975	<0.1%	0%	0.20%	0%	0%	0%	0%	0%	0%

For peer review only

Supplementary information 6: Lorenz curve

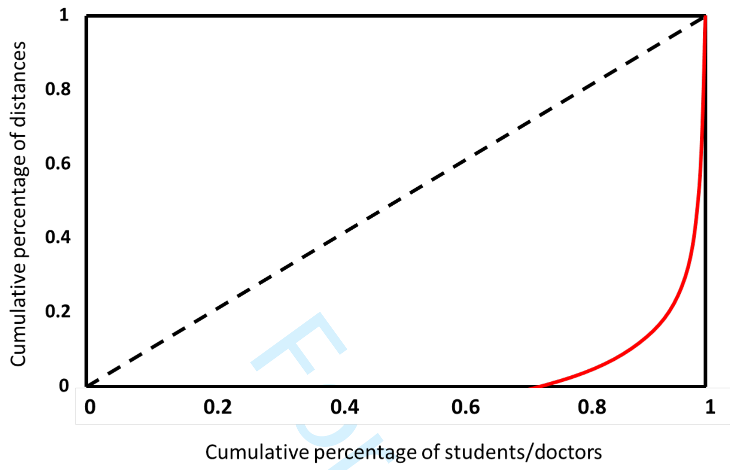

Figure S1: Lorenz curve of the Leslie Matrix. Equality (Gini coefficient equal to 0) is achieved when the Lorenz curve (red line) coincide with the diagonal (dashed). The diagonal represents the status where all distances have equal number of students. In this figure a strong inequality is shown, with the entire Lorenz curve laying on values above 0.7.

For peer review only

Reporting checklist for cohort study.

Based on the STROBE cohort guidelines.

Instructions to authors

Complete this checklist by entering the page numbers from your manuscript where readers will find each of the items listed below.

Your article may not currently address all the items on the checklist. Please modify your text to include the missing information. If you are certain that an item does not apply, please write "n/a" and provide a short explanation.

Upload your completed checklist as an extra file when you submit to a journal.

In your methods section, say that you used the STROBE cohortreporting guidelines, and cite them as:

von Elm E, Altman DG, Egger M, Pocock SJ, Gotzsche PC, Vandenbroucke JP. The Strengthening the Reporting of Observational Studies in Epidemiology (STROBE) Statement: guidelines for reporting observational studies.

		Reporting Item	Page Number
**Title and abstract**			
Title	#1a	Indicate the study's design with a commonly used term in the title or the abstract	1
Abstract	#1b	Provide in the abstract an informative and balanced summary of what was done and what was found	3
**Introduction**			
Background / rationale	#2	Explain the scientific background and rationale for the investigation being reported	4
Objectives	#3	State specific objectives, including any prespecified hypotheses	4
**Methods**			
Study design	#4	Present key elements of study design early in the paper	5
Setting	#5	Describe the setting, locations, and relevant dates, including periods of recruitment, exposure, follow-up, and data	5

For peer review only

		collection	
Eligibility criteria	#6a	Give the eligibility criteria, and the sources and methods of selection of participants. Describe methods of follow-up.	n/a no selection
Eligibility criteria	#6b	For matched studies, give matching criteria and number of exposed and unexposed	n/a no matched study
Variables	#7	Clearly define all outcomes, exposures, predictors, potential confounders, and effect modifiers. Give diagnostic criteria, if applicable	6 and 7
Data sources / measurement	#8	For each variable of interest give sources of data and details of methods of assessment (measurement). Describe comparability of assessment methods if there is more than one group. Give information separately for for exposed and unexposed groups if applicable.	5
Bias	#9	Describe any efforts to address potential sources of bias	2
Study size	#10	Explain how the study size was arrived at	n/a all database considered
Quantitative variables	#11	Explain how quantitative variables were handled in the analyses. If applicable, describe which groupings were chosen, and why	5 and 6
Statistical methods	#12a	Describe all statistical methods, including those used to control for confounding	
5 and 6			
Statistical methods	#12b	Describe any methods used to examine subgroups and interactions	5 and 6
Statistical methods	#12c	Explain how missing data were addressed	n/a no missing data
Statistical methods	#12d	If applicable, explain how loss to follow-up was addressed	n/a
Statistical methods	#12e	Describe any sensitivity analyses	
n/a Different models were tested (supplementary)			

For peer review only

Results

Participants	#13a	Report numbers of individuals at each stage of study—eg numbers potentially eligible, examined for eligibility, confirmed eligible, included in the study, completing follow-up, and analysed. Give information separately for for exposed and unexposed groups if applicable.	n/a
Participants	#13b	Give reasons for non-participation at each stage	n/a
Participants	#13c	Consider use of a flow diagram	
n/a			
Descriptive data	#14a	Give characteristics of study participants (eg demographic, clinical, social) and information on exposures and potential confounders. Give information separately for exposed and unexposed groups if applicable.	n/a
Descriptive data	#14b	Indicate number of participants with missing data for each variable of interest	
n/a			
Descriptive data	#14c	Summarise follow-up time (eg, average and total amount)	
n/a			
Outcome data	#15	Report numbers of outcome events or summary measures over time. Give information separately for exposed and unexposed groups if applicable.	
9			
Main results	#16a	Give unadjusted estimates and, if applicable, confounder-adjusted estimates and their precision (eg, 95% confidence interval). Make clear which confounders were adjusted for and why they were included	n/a Supplementary file
Main results	#16b	Report category boundaries when continuous variables were categorized	n/a
Main results	#16c	If relevant, consider translating estimates of relative risk into absolute risk for a meaningful time period	
n/a			

For peer review only

Other analyses	#17	Report other analyses done—eg analyses of subgroups and interactions, and sensitivity analyses	n/a Supplementary file
**Discussion**			
Key results	#18	Summarise key results with reference to study objectives	10 and 11
Limitations	#19	Discuss limitations of the study, taking into account sources of potential bias or imprecision. Discuss both direction and magnitude of any potential bias.	2
Interpretation	#20	Give a cautious overall interpretation considering objectives, limitations, multiplicity of analyses, results from similar studies, and other relevant evidence.	10 and 11
Generalisability	#21	Discuss the generalisability (external validity) of the study results	10 and 11
**Other Information**			
Funding	#22	Give the source of funding and the role of the funders for the present study and, if applicable, for the original study on which the present article is based	12

Notes:

- 6a: n/a no selection
- 6b: n/a no matched study
- 10: n/a all database considered
- 12c: n/a no missing data
- 12e: n/a Different models were tested (supplementary)
- 16a: n/a Supplementary file
- 17: n/a Supplementary file The STROBE checklist is distributed under the terms of the Creative Commons Attribution License CC-BY. This checklist was completed on 06. July 2023 using https://www.goodreports.org/, a tool made by the EQUATOR Network in collaboration with Penelope.ai

For peer review only
